# The Cytotoxic Necrotizing Factor of *Yersinia pseudotuberculosis* (CNF_Y_) Enhances Inflammation and Yop Delivery during Infection by Activation of Rho GTPases

**DOI:** 10.1371/journal.ppat.1003746

**Published:** 2013-11-07

**Authors:** Janina Schweer, Devesha Kulkarni, Annika Kochut, Joern Pezoldt, Fabio Pisano, Marina C. Pils, Harald Genth, Jochen Huehn, Petra Dersch

**Affiliations:** 1 Department of Molecular Infection Biology, Helmholtz Centre for Infection Research, Braunschweig, Germany; 2 Department of Experimental Immunology, Helmholtz Centre for Infection Research, Braunschweig, Germany; 3 Mouse Pathology, Animal Experimental Unit, Helmholtz Centre for Infection Research, Braunschweig, Germany; 4 Institute for Toxicology, Medical School Hannover, Hannover, Germany; Stony Brook University, United States of America

## Abstract

Some isolates of *Yersinia pseudotuberculosis* produce the cytotoxic necrotizing factor (CNF_Y_), but the functional consequences of this toxin for host-pathogen interactions during the infection are unknown. In the present study we show that CNF_Y_ has a strong influence on virulence. We demonstrate that the CNF_Y_ toxin is thermo-regulated and highly expressed in all colonized lymphatic tissues and organs of orally infected mice. Most strikingly, we found that a *cnfY* knock-out variant of a naturally toxin-expressing *Y. pseudotuberculosis* isolate is strongly impaired in its ability to disseminate into the mesenteric lymph nodes, liver and spleen, and has fully lost its lethality. The CNF_Y_ toxin contributes significantly to the induction of acute inflammatory responses and to the formation of necrotic areas in infected tissues. The analysis of the host immune response demonstrated that presence of CNF_Y_ leads to a strong reduction of professional phagocytes and natural killer cells in particular in the spleen, whereas loss of the toxin allows efficient tissue infiltration of these immune cells and rapid killing of the pathogen. Addition of purified CNF_Y_ triggers formation of actin-rich membrane ruffles and filopodia, which correlates with the activation of the Rho GTPases, RhoA, Rac1 and Cdc42. The analysis of type III effector delivery into epithelial and immune cells *in vitro* and during the course of the infection further demonstrated that CNF_Y_ enhances the Yop translocation process and supports a role for the toxin in the suppression of the antibacterial host response. In summary, we highlight the importance of CNF_Y_ for pathogenicity by showing that this toxin modulates inflammatory responses, protects the bacteria from attacks of innate immune effectors and enhances the severity of a *Yersinia* infection.

## Author Summary

Various toxins and effector proteins of bacterial pathogens have been found to manipulate eukaryotic cell machineries to promote persistence and proliferation within their hosts. Many of these virulence factors target small Rho GTPases, but their role in pathogenesis is often unknown. Here, we addressed the expression and functional consequences of the CNF_Y_ toxin found in some isolates of *Y. pseudotuberculosis*. We found that CNF_Y_ besides modulating the cell cytoskeleton by activation of the GTPases RhoA, Rac1 and Cdc42, contributes to increased inflammation and tissue damage. Moreover, CNF_Y_ increases the ability of *Yersinia* to prevent the attack of the immune system, by enhancing the delivery of antiphagocytic and cytotoxic effectors into professional phagocytes. Our findings provide the first insights into the multi-functional action and severe consequences of the CNF_Y_ toxin on the inflammatory response and disease-associated tissue damage during the natural course of the infection.

## Introduction

Enteropathogenic *Yersinia* species such as *Y. enterocolitica* and *Y. pseudotuberculosis* initially infect the terminal ileum and colonize the Peyer's patches (PPs) within several hours of infections. Bacteria are subsequently transported to the mesenteric lymph nodes (MLNs) and can also spread systemically to reach liver and spleen via the bloodstream. The infections typically result in enteritis, enterocolitis and mesenteric lymphadenitis where the infected tissues show formation of microabscesses or granuloma-like lesions with central necrosis [1].

Enteropathogenic *yersiniae* have been shown to secrete exotoxins and/or inject effector proteins by specialized secretion machineries to manipulate host cell functions, including cytoskeletal rearrangements, to prevent immune responses and to establish a successful infection. They encode a type III secretion system (T3SS) on a 70 kb virulence-associated plasmid (pYV) that is essential for their defense against the host immune system [2]–[4]. The *Yersinia* T3SS has been shown to form a syringe-like apparatus with a thin needle-like surface exposed projection [5]. It is used to insert a translocation channel (composed of YopB and YopD) within the host membrane to inject the effector proteins YopE, YopH, YopJ/YopP, YopK/YopQ, YopM, YopO/YpkA, and YopT into the cell's cytoplasm. Yops target different cell signaling molecules and processes, in particular cytokine production and actin dynamics, often resulting in the inhibition of phagocytosis [6].

YopH is a tyrosine phosphatase that dephosphorylates proteins of the focal adhesion complex [7]–[10]. The effectors YopE, YopT and YopO/YpkA manipulate the regulation of Rho GTPases, which control the formation of lamellipodia, filopodia and stress fibers [2], [11]. YopJ/YopP promotes cell death of macrophages by inactivating the counterregulators of the Toll-like receptor 4-triggered apoptotic pathway, the mitogen-activated protein kinase kinases (MEKs) and the inhibitor κB kinase β (IKK β) [12]–[16]. YopM forms a complex with RSK and PRK kinase isoforms, traffics to the nucleus, and is important for *Yersinia* to persist in liver and spleen with a contextual decrease of several proinflammatory cytokines, including IL-1β, IL-12, IL-18, interferon γ, and TNF-α, and depletion of NK cells [17]–[21]. The effector YopK/YopQ seems to play a role in orchestrating the translocation of effector proteins by modulating the ratio of the pore-forming proteins YopB and YopD. This appears to prevent unintended Yop delivery and neutrophil death, which would enhance the inflammatory response possibly favoring the host [22]–[24].

Insertion of the YopB/D translocation channel allows Yop delivery while maintain the host cell membrane intact. The YopB/D complex results in activation of Rho GTPases, actin polymerization and pore-formation. However, pore formation is usually prevented by the GTPase-downregulating function of YopE and YopT. Yet, expression of constitutively active forms of Rac1 and RhoA leads to a loss of membrane integrity and results in increased pore formation even when YopE and YopT are expressed [25]. In addition, signaling pathways triggered by high affinity-binding of the main *Yersinia* adhesins YadA and InvA to β_1_ integrin receptors and YopB/D signaling were shown to induce activity of Rho GTPases and actin polymerization which are crucial for efficient translocation of the Yop effectors [26].

Another *Yersinia* factor shown to activate the small GTPase RhoA is the cytotoxic necrotizing factor-Y (CNF_Y_) [27], [28]. CNF_Y_ is prevalent in some *Y. pseudotuberculosis* isolates, e.g. the widely used *Y. pseudotuberculosis* strain YPIII. All these strains belong to the serogroup III, but other isolates of this serogroup do not express CNF_Y_ and contain deletions within the corresponding *cnfY* gene [27].

On the amino acid level, CNF_Y_ is highly similar (>68%) to the CNF toxins found mainly in *E. coli* strains isolated from patients and domestic animals with extraintestinal infections (CNF1-3) [29], [30]. CNF1 is the best-characterized toxin of this class of bacterial toxins and is transferred to host cells through outer membrane vesicles (OMVs) [31]–[34]. The CNF1 toxin is a single-chain A-B toxin with an N-terminal delivery domain including subdomains for receptor binding, pore formation and proteolytic cleavage, and a C-terminal deamidase domain [35], [36]. Internalization of the toxin into target cells occurs through receptor-mediated endocytosis, which appears to be independent of clathrin and lipid rafts (sphingolipid/cholesterol rich microdomains) [37], [38]. After uptake, the 55 kDa C-terminal deamidase domain is autocatalytically cleaved off in the late endosome, and delivered into the cytoplasm in a pH-dependent manner [39].

CNF1 deamidates Gln-61/-63 of RhoA, Rac1 and Cdc42 to Glu-61/-63 resulting in Rho GTPases with a blocked GTP hydrolase activity. Deamidated Rho GTPases induces polymerization of F-actin at focal contacts, increase cell-matrix adhesion, and promote formation of stress fibers, lamellipodia and filopodia, which led to the classification as ‘constitutively active’ [40]–[45]. Cytoskeletal rearrangements attributed to CNF lead to multinucleated cells due to inhibited cytokinesis with ongoing cell cycle progression [46]. Additionally, CNF1 has been reported to (i) induce phagocytosis in epithelial cells and reduce CR3-mediated phagocytosis in monocytes [47], [48], (ii) promote bacterial cell entry [49], (iii) decrease the barrier function of intestinal tight junctions [40], [50], (iv) decrease transmigration of polymorphonuclear leukocytes across a T84 monolayer [51], and (v) induce apoptosis of bladder cells [52].

The overall amino acid sequence of CNF_Y_ of *Y. pseudotuberculosis* is very similar to CNF1. However, CNF_Y_ is not recognized by neutralizing antibodies against CNF1 [27]. Moreover, CNF_Y_ seems to bind to different cell receptors and preferentially deamidates RhoA (over Rac1 and Cdc42) in cultured epithelial cells [28], [38]. Although CNF_Y_ and certain Yop effectors alter the cytoskeleton by affecting the activity of the Rho GTPases, little is known about the interplay, cooperation and joint role of these toxins in the pathogenic lifestyle of *Y. pseudotuberculosis*. Here, we provide evidence that CNF_Y_ is an important virulence factor of *Y. pseudotuberculosis* YPIII. CNF_Y_ is shown to enhance Yop protein delivery, which is crucial for pathogenicity. Furthermore, the toxin was found to induce inflammatory responses and increase the severity of a *Yersinia* infection.

## Results

### CNF_Y_ is expressed in all infected tissues and organs throughout the infection

Since many *Y. pseudotuberculosis* isolates as well as *Y. pestis* contain deletions within the *cnfY* gene [27], we first tested whether the intact *cnfY* toxin gene in the *Y. pseudotuberculosis* wild-type strain YPIII is expressed and induced under virulence-relevant growth conditions. A *cnfY-lacZ* transcriptional fusion was only slightly expressed when *Y. pseudotuberculosis* was grown at 25°C, but its expression was strongly induced at 37°C and reached its maximum during stationary phase (Fig. **S1A**). High *cnfY* transcription was generally observed in complex media, in particular BHI, whereas only low expression levels were detected in all tested minimal media (Fig. **S1B**, data not shown). In summary, *cnfY* is predominantly expressed at 37°C in a nutrient rich environment, resembling conditions found in the mammalian intestinal tract.

**Figure 1:**
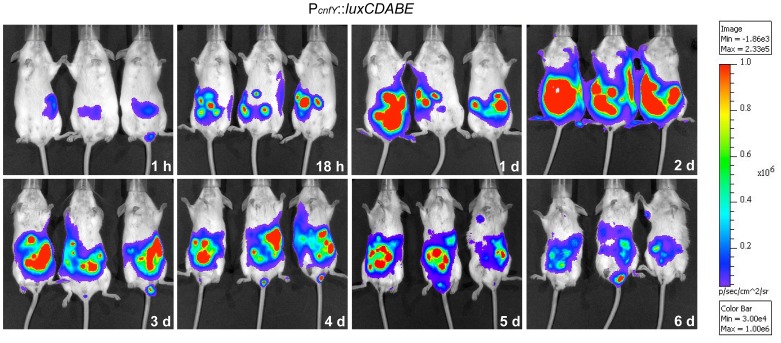
*In vivo* expression analysis of the *cnfY-luxCDABE* fusion. 2×10^8^ bacteria of *Y. pseudotuberculosis* YPIII pJNS02 (P*_cnfY_::luxCDABE*) was used to orally infect BALB/c mice. At indicated time points, mice were anesthesized and bioluminescence was detected with a CCD camera (*in vivo* imaging system) on the ventral side.

This result prompted us to test expression of the toxin during infection. BALB/c mice were orally infected with 2×10^8^ bacteria of the *Y. pseudotuberculosis* wild-type strain YPIII expressing a *cnfY-luxCDABE* fusion, and the bioluminescent signal was monitored in the mice for six days using an *in vivo* imaging system. Only very low luciferase activity was measured in the bacterial culture before infection (data not shown) and in the intestinal tract directly after oral ingestion (1 h, Fig. **1**). However, a very strong bioluminescent signal of the *cnfY-luxCDABE* fusion was detectable during the entire following course of the infection. The most intensive signals were detected two days post infection in the intestine and associated lymphoid tissues (Fig. **1**). No light emission was monitored in mice infected with bacteria carrying the promoterless *luxCDABE* operon in the identical expression system (data not shown).

**Figure 2:**
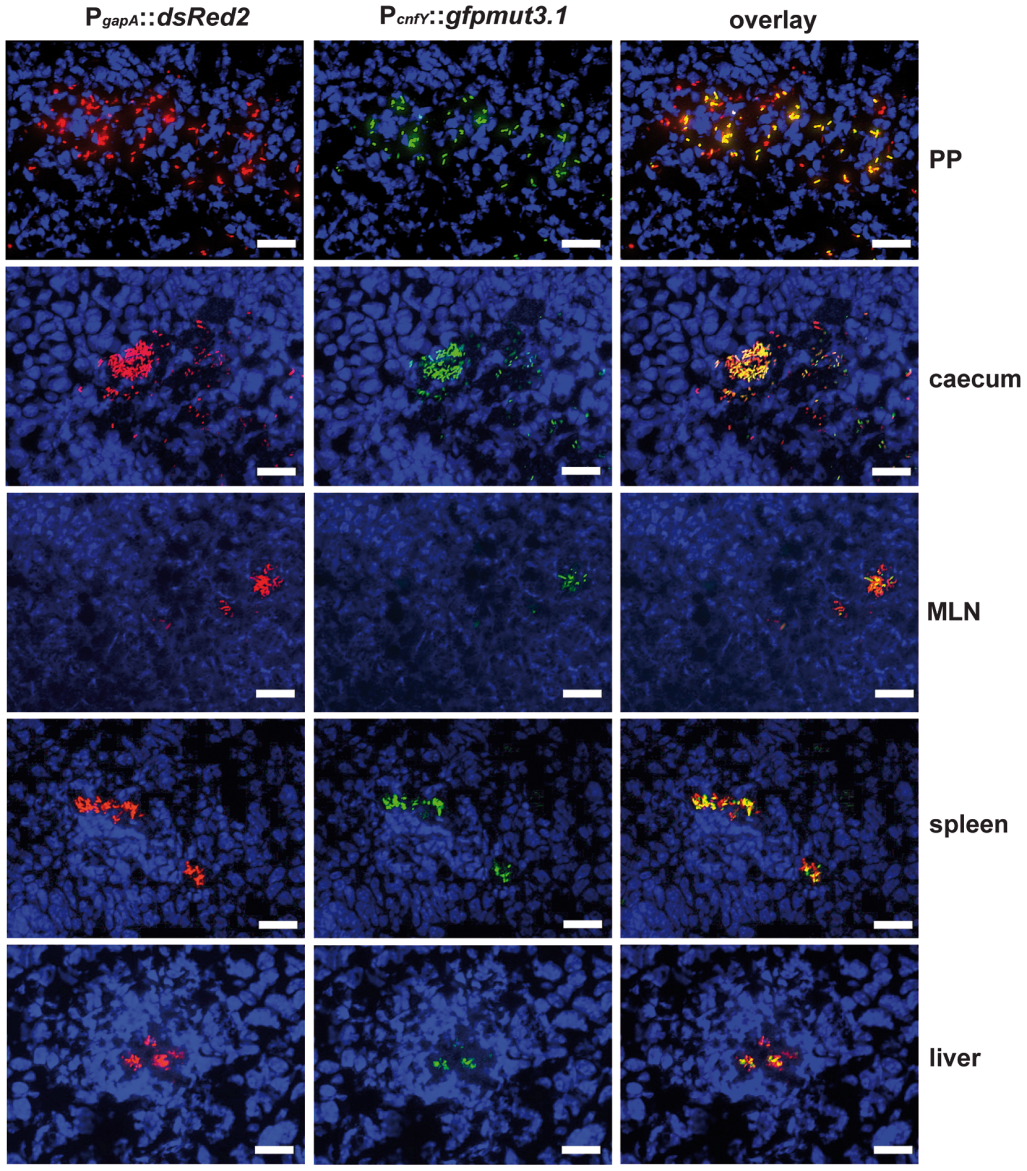
Expression of P*_cnfY_*::*gfp* in the lymphatic tissues and organs. 2×10^8^ bacteria of *Y. pseudotuberculosis* YPIII pFU228/pJNS03 (P*_gapA_*::dsred, P*_cnfY_::gfpmut3.1*) were used to orally infect BALB/c mice. Three days post infection, mice were sacrificed, and PPs, caecum, MLNs, spleen and liver were isolated. Histological slides were prepared and analyzed by fluorescence microscopy to detect bacteria in the tissues by expression of the reporter protein DsRed2. In parallel *cnfY* expression in the bacteria was monitored by GFPmut3.1 mediated fluorescence. White bars indicate 20 µm.

In order to study *cnfY* expression in the individual infected tissues, we used a set of established fluorescent fusion vectors for *in vivo* expression analysis. To do so, *Y. pseudotuberculosis* YPIII harboring a plasmid-encoded constitutive P*_gapA_*::*dsred2* reporter construct and a compatible P*_cnfY_*::*gfp*_mut3.1_ fusion was used to infect BALB/c mice. Five days post infection, the small intestine, caecum, colon, PPs, MLNs, spleen and liver were isolated and cryosections were prepared. The bacteria in the tissues were visualized by monitoring dsRed2, and then tested for P*_cnfY_*::*gfp*_mut3.1_. As shown in Fig. **2**, the P*_cnfY_*::*gfp*_mut3.1_ fusion was expressed in all tested organs. In summary, a temperature shift to 37°C, but most likely no tissue-specific signals are required to induce toxin expression in infected tissues.

### CNF_Y_ is crucial for virulence of *Y. pseudotuberculosis* YPIII

**Figure 3:**
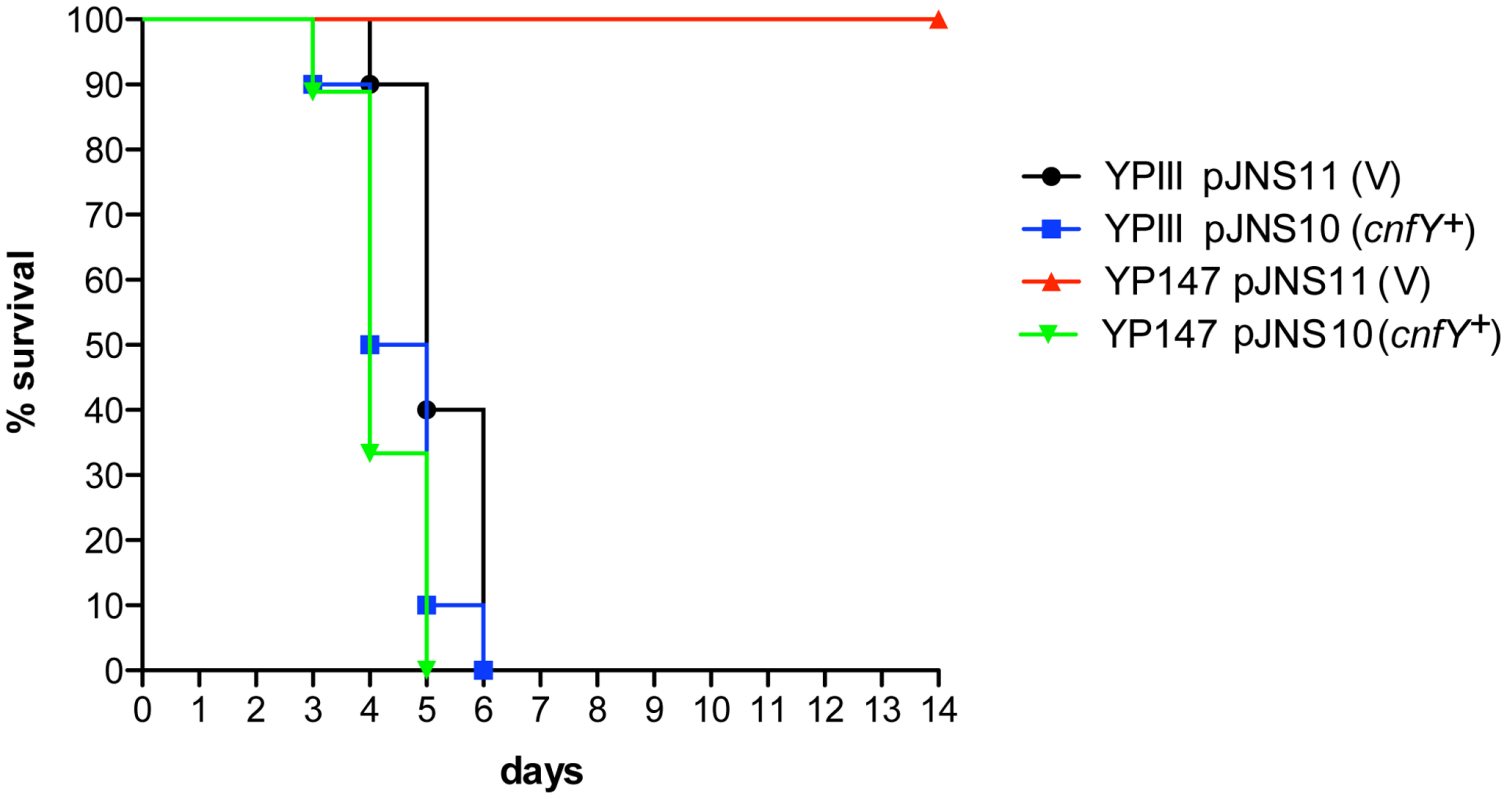
Influence of *cnfY* on the survival of BALB/c mice infected with *Y. pseudotuberculosis*. Survival of BALB/c mice (n = 10/strain) were monitored up to 14 days after oral infection with 2×10^9^ cfu of *Y. pseudotuberculosis* YPIII (black line), the *cnfY* mutant YP147 (red line) harbouring the empty vector pJNS11, and the strains YPIII pJNS10 (*cnfY* ^+^) (green line) and YP147 pJNS10 (*cnfY* ^+^) (blue line).

Absence of a functional toxin gene in other *Y. pseudotuberculosis* clinical isolates, may suggest that CNF_Y_ only adds another potential virulence factor to the variety of effector proteins and toxins that are produced by this pathogen. However, high expression of *cnfY* during the entire course of an infection also indicates that presence of this toxin may enhance the pathogenicity of *Y. pseudotuberculosis*. To first assess the impact of CNF_Y_ on pathogenesis, the potential of the *Y. pseudotuberculosis* wild-type strain YPIII and the isogenic *cnfY*-deficient strain to cause lethal infections was compared. BALB/c mice were orally infected with 2×10^9^ bacteria of the *cnfY* mutant (YP147) and the wild-type strain (YPIII) harboring the empty vector (pJNS11) or a *cnfY*-encoding plasmid (pJNS10). Survival and weight of the mice were monitored over two weeks and date of death was recorded (Fig. **3**, **S2**). Mice infected with YPIII showed signs of the infection, e.g. weight loss, piloerection and lethargy, and succumbed to infection between day four and day six. Strikingly, none of the mice infected with YP147 developed severe disease symptoms and all mice were still alive 14 days post infection. Monitoring of body weight demonstrated that also mice infected with the *cnfY* knock-out strain YP147 showed a slight reduction in weight, but they recovered quickly and regained weight (Fig. **S2**). Presence of the *cnfY*-encoding low-copy number plasmids reverted the avirulent phenotype of the *cnfY* mutant and reduced the average day of death of the wild-type strain YPIII by one day, most likely due to the overexpression of the toxin. The *Y. pseudotuberculosis* YPIII isolate, unlike other *Y. pseudotuberculosis* strains, is unable to replicate in murine macrophages due to a defective allele of *phoP* [53]. To exclude that CNF_Y_ influence on virulence is only visible in a *phoP*-deficient derivative with an overall lower pathogenicity, the inability to grow in macrophages was complemented by an exchange of the allele against the *phoP* ORF from *Y. pseudotuberculosis* IP32953. However, when mice were challenged with 2×10^9^ CFU of the equivalent *phoP* ^+^ strains, 100% of the mice infected with the CNF_Y_-positive strain died during the observation period, while 80% of the mice infected with the isogenic *cnfY*-deficient strain survived and regained weight (Fig. **S3**).

**Figure 4:**
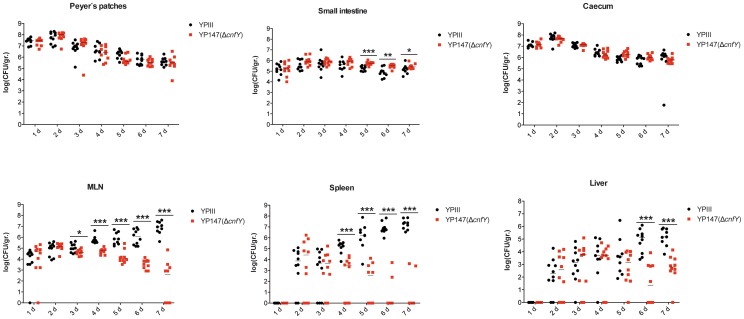
Influence of *cnfY* on the virulence of *Y. pseudotuberculosis*. BALB/c mice were infected intragastrically with an inoculum of 2×10^8^ cfu of *Y. pseudotuberculosis* wild-type YPIII or the *cnfY* mutant YP147. After 1–7 days of infection, mice were sacrificed and the number of bacteria in homogenized host tissues and organs (PPs, caecum, small intestine, MLNs, spleen, liver) was determined by plating. Data of two independent experiments (5 mice/group) are represented in scatter plots of numbers of CFU per gram as determined by counts of viable bacteria on plates. The statistical significances between the wild-type and the *cnfY* mutant were determined by the Mann-Whitney test. P-values: *: <0.05; **: <0.01 ***: <0.001.

To gain a deeper insight into the differences in the infection process of CNF_Y_-positive and -negative strains, we determined the number of bacteria that colonized the small intestine, caecum, PPs, MLNs, liver and spleen of BALB/c mice at different time points after oral infection with 2×10^8^ bacteria (Fig. **4**). Comparable amounts of wild-type (YPIII) and the mutant strains (YP147) were recovered from PPs and caecum during infection, and only a very small increase of bacterial counts was observed with the *cnfY* mutant in the small intestine at days 5–7 post infection (Fig. **4**). However, significantly reduced numbers of YP147 were recovered from MLNs and spleen (Fig. **4**). The number of *cnfY*-positive and -negative bacteria in these organs was almost identical up to day three post infection, but the *cnfY* mutant was eliminated very rapidly later during the infection. At day seven, none or only few mutants were recovered from MLNs and spleen, whereas 10^8^–10^9^ bacteria of the wild-type strain were recovered per gram of both organs. The effect was less pronounced in the liver, but the strongly reduced number of mutant bacteria relative to the wild-type bacteria six and seven days after infection clearly indicated that the presence of CNF_Y_ is also advantageous for the colonization of the liver (Fig. **4**). This demonstrated that loss of CNF_Y_, resulting in avirulence of *Y. pseudotuberculosis* YPIII, is reflected by a fast elimination of the bacteria from MLNs, liver and spleen.

Within the first week after infection with wild-type strain YPIII the size of the spleen and liver decreased two-fold, whereby changes of the organ size were first visible at day three post infection (Fig. **S4A**,**B**). In contrast, infection with the isogenic *cnfY* mutant strain YP147 had no effect on the size of the liver and induced a considerable increase of the size of the spleen. In addition, mice infected with wild-type strain YPIII had significantly shorter intestines (30%) at day six and seven post infection than mice infected with the *cnfY* mutant (Fig. **S4C**). The shortening of the intestine is a sign of marked intestinal inflammation. This indicated that CNF_Y_ not only affects colonization of systemic organs, but has also a strong influence on the host's inflammatory response against the bacterial infection.

**Figure 5:**
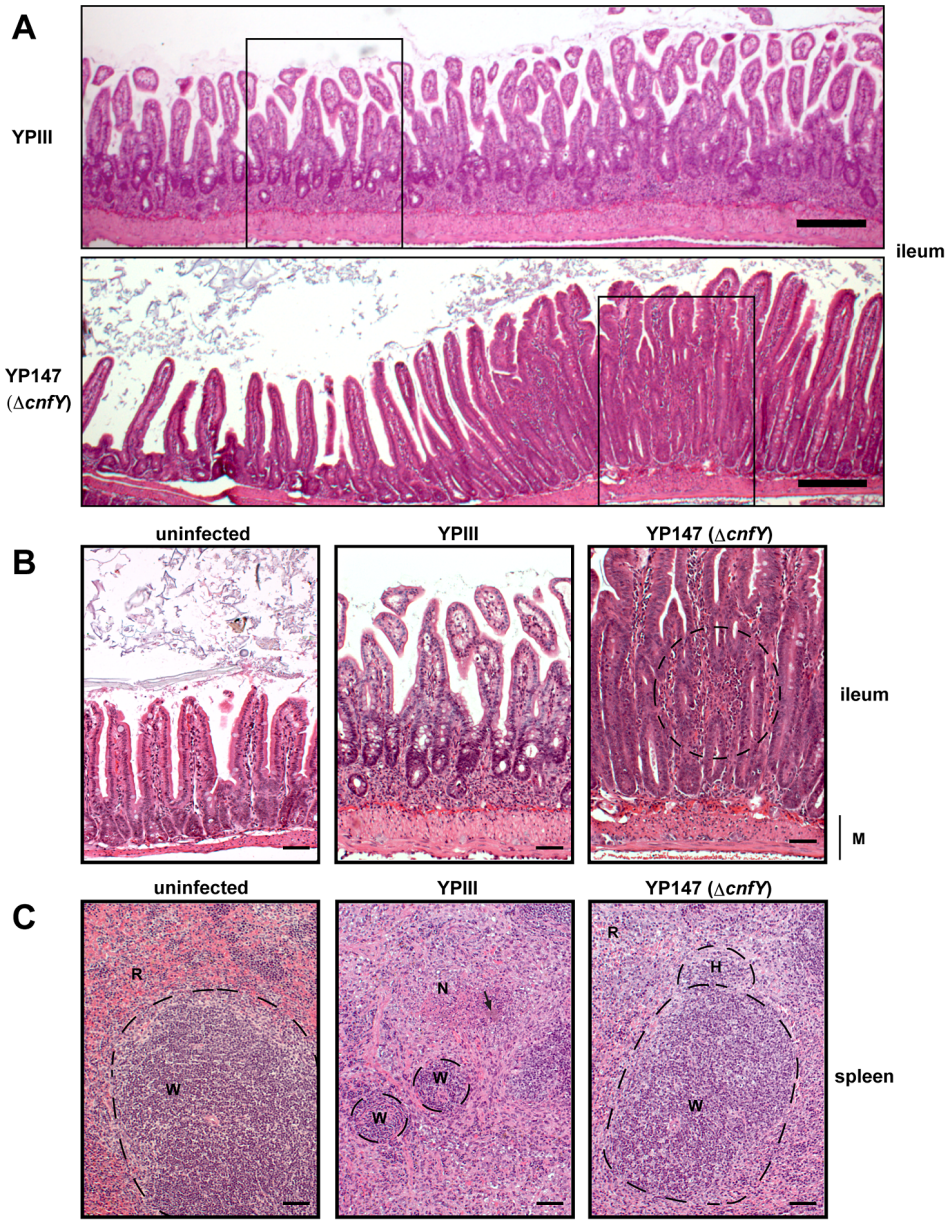
Histology of infected organs. Histopathology of H & E stained sections of the ileum and the spleen of mice orally infected with 2×10^8^ bacteria of YPIII and YP147. (**A**) ileum (6 days post infection); YPIII: diffuse invasion of inflammatory cells into the lamina propria. YP147: Focal invasion of inflammatory cells into the lamina propria; adjacent tissue was unaffected. Bar represents 200 µm. White boxes indicate magnified areas in the slides below. (**B**) Magnified section of the ileum of an uninfected mouse (left panel), a mouse infected with YPIII - magnification of the ileum selection shown in **A** (middle panel), and a mouse infected with YP147- magnification of the focal invasion of inflammatory cells (right panel), local inflammation is indicated by the circle. Bar represents 50 µm. (**C**) spleen (6 days post infection); uninfected mouse (left panel). YPIII infected mouse: splenic atrophy and bacterial colony surrounded by focal necrosis. Pictures shown are representatives of multiple fields and samples of 9 mice. Arrow points to the bacterial foci (middle panel). YP147: hyperplasia of the white pulp and activated lymphoid follicle (right panel). W: white pulp indicated by dashed lines, N: necrosis, H: hyperplasia, R: red pulp, M: muscularis mucosa. Bar represents 50 µm.

Histopathological examination of the infected host tissues demonstrated marked differences of the overall inflammatory reaction, which was stronger in YPIII-infected animals, especially in the small intestine and spleen compared to YP147-infected mice. In the intestine, inflammation was most prominent in the ileum and caecum in both groups. However, in YPIII-infected mice inflammation was diffuse affecting the entire ileum at day six (Fig. **5A**, upper panel, **5B** middle panel). In YP147-infected mice inflammation was locally restricted to multifocal lesions characterized by the presence of inflammatory cells from the muscular layer up to the epithelial cells (Fig. **5A**, lower panel, **5B** right panel). In these areas inflammation led to epithelial cell hyperplasia (increased proliferation) resulting in an increase of the villi length. However, this lesion is only locally restricted and adjacent tissue remains unaltered. In addition, inflammation was more generalized in YPIII- compared to YP147-infected organs. In mice infected with YP147, no bacterial foci (diffuse patches of bacteria) could be detected microscopically in hematoxylin and eosin (H & E) stained sections of the spleen at day six post infection, whereas in the majority of YPIII-infected mice bacterial foci were visible in the histological sections (Fig. **5C**). YPIII infections were accompanied by a more severe inflammation of the spleen, where presence of the bacteria resulted in necrotizing spleenitis leading to splenic atrophy with marked depletion of the white pulp. YPIII caused multifocal necrosis in spleen, whereas in YP147-infected spleens, only mild hyperplasia of the white pulp and increased erythropoiesis were found (Fig. **5C**). Taken together, CNF_Y_ has a significant influence on the number of microcolonies in the tissues and leads to a more severe and widespread inflammation in the small intestine, liver and spleen.

### CNF_Y_ modulates the innate immune response

**Figure 6:**
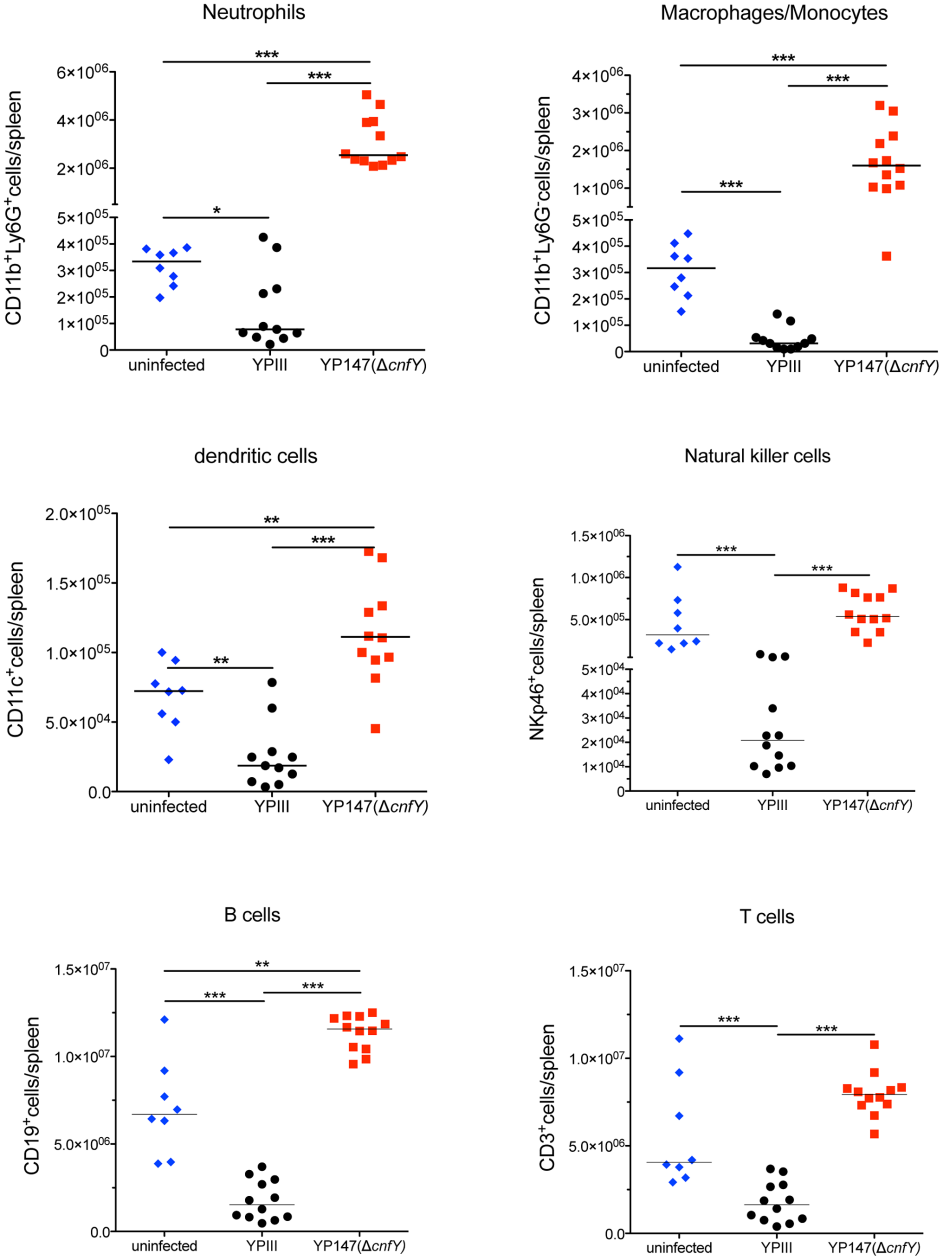
Analysis of immune cells recruited to the spleen after infection with *Y. pseudotuberculosis* YPIII or YP147. About 2×10^8^ bacteria (YPIII, YP147) were used to infect BALB/c mice. Three days after infection, mice were sacrificed, the spleens were isolated, homogenized and the cell suspensions were used for flow cytometric analysis. Values on the y axis indicate the numbers of cells isolated from spleen infected with the wild-type strain YPIII or the *cnfY* mutant strain YP147. CD11b^+^/Ly6G^−^: macrophages/monocytes; CD11b^+^/Ly6G^+^: neutrophils; CD11c^+^: DCs; NKp46^+^: NK cells, CD3^+^: T cells, CD19^+^: B cells. The data show the median from at least two different experiments each done with groups of 4–6 mice. The asterisks indicates that there was a significant difference in the number of the indicated cells type in the whole organ in YP147-infected compared to YIPIII-infected mice based on a Mann-Whitney test with * (P<0.05), ** (P<0.01) and *** (P<0.001).

Because of the strong influence of CNF_Y_ on the colonization of bacteria in MLNs, spleen and liver, it was hypothesized that the toxin might counteract host immune defenses. To test this hypothesis, we infected BALB/c mice with 2×10^8^ bacteria of the wild-type or the *cnfY* mutant strain, and immune cell composition in the spleen was analyzed by multi-color flow cytometry three days and six days post infection. The spleen was chosen since here the most pronounced CNF_Y_-triggered pathological effects had been observed. Cell suspensions of isolated tissues were prepared and cells were stained with fluorescently labeled antibodies to distinguish neutrophils from macrophages/monocytes, dendritic cells (DCs), natural killer (NK) cells, B cells, and T cells (Fig. **S5**). All alterations of immune cell populations seen at day six (data not shown) were already visible at day three post infection, when the bacterial load is still similar and the overall health status of YPIII-infected mice is only slightly and not severely reduced as at day six. A very pronounced variation of the immune cell population between the YPIII- and YP147-infected mice was observed (Fig. **6**). All types of immune cells were significantly decreased in the spleen three days after infection with YPIII when the spleen started to shrink, but the most severe changes were observed with cells of the innate immune system. In particular, numbers of macrophages, monocytes and NK cells were significantly reduced; whereas reduction of neutrophils and conventional DCs was less pronounced. In contrast, no reduction of immune cells was detectable in spleens of YP147-infected mice (Fig. **6**). In contrast, a significant higher influx of neutrophils and macrophages/monocytes was observed, which is consistent with the rapid clearance of mutant bacteria from the spleen upon triggering of the immune response. To determine whether CNF_Y_ affects the steady-state level of certain cell populations, the population percentage was also compared and further confirmed a significant expansion of neutrophils and macrophages/monocytes in YP147-infected spleens (Fig. **S6**). These and the histopathological data strongly suggest that the CNF_Y_ toxin reduces influx and/or causes rapid cell death of invading immune cells in the spleen.

### CNF_Y_ enhances Yop delivery into macrophages

Our infection experiments clearly demonstrated that absence of the CNF_Y_ toxin renders the bacteria completely avirulent, resulting in the clearance of the bacteria in MLNs, liver and spleen. A similar attenuation in mouse models of oral infection was observed (i) when the virulence plasmid, encoding the T3SS and the Yop effectors is cured from *Y. pseudotuberculosis* YPIII, (ii) when multiple *yop* genes were deleted or (iii) when the regulator LcrF that controls expression of the T3SS/Yops is absent [54], [55]. Moreover, a significant influx of neutrophils was observed in the spleen of mice infected with a *yopM* mutant strain of *Y. pestis*, while the numbers of neutrophils decreased during infection with the parental strain [20], [21]. In addition, YopJ translocation has been shown to promote cell death of professional phagocytes [13], [15]. This suggested that the CNF_Y_ toxin is important for the efficient injection of the Yop effectors into host cells during the infection process. In fact, recent work by Mejia *et al.* [26] demonstrated that efficient translocation of the Yop effectors requires Rho activation – a process that has been shown to be stimulated by the CNF_Y_ toxin [28], [38].

**Figure 7:**
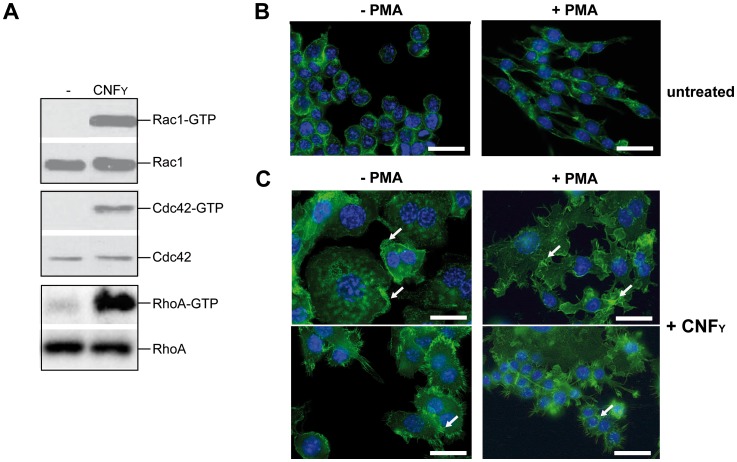
CNF_Y_-mediated activation of Rho GTPases and morphologic modification and F-actin rearrangements of murine macrophages. (**A**) J774A.1 macrophages were treated with 25 nM purified CNF_Y_ toxin for 3 h. Toxin treated cells were lysed and aliquots of the cell lysates were taken for westernblot analysis. Remaining samples were incubated with beads-coupled with the GTPase-binding domains of effector proteins interacting with GTP-bound Cdc42, Rac1 and RhoA. The amount of the three different activated Rho GTPases and the total amount of the Rho GTPases of the lysates were analyzed with specific antibodies. (**B,C**) Fluorescence microscopy of unstimulated (−PMA) and stimulated (+PMA for 48 h) J774A.1 macrophages untreated (**B**) or treated with 10 nM purified CNF_Y_ toxin (**C**) for 24 h and stained with phalloidin-FITC and DAPI. The arrows indicate actin-rich membrane ruffles, filopodia and stress fibres induced by CNF_Y_ incubation.

To address whether CNF_Y_-mediated activation of Rho GTPases influences Yop-translocation into professional phagocytes, we first tested the influence of recombinant CNF_Y_ toxin on non-activated and PMA-activated macrophages, thus mimicking its effect on unstimulated and stimulated macrophages during infection. Intoxification of murine macrophages (J774A.1) led to activation of all three Rho GTPases, RhoA, Cdc42 and Rac1 (Fig. **7A**). CNF_Y_ further induced a marked increase in cell size with some giant multinucleated cells (Fig. **7B**). These CNF_Y_ effects occurred independently of macrophage stimulation with PMA. This indicates that CNF_Y_ controls actin dynamics in macrophages through deamidation of Rho GTPases.

**Figure 8:**
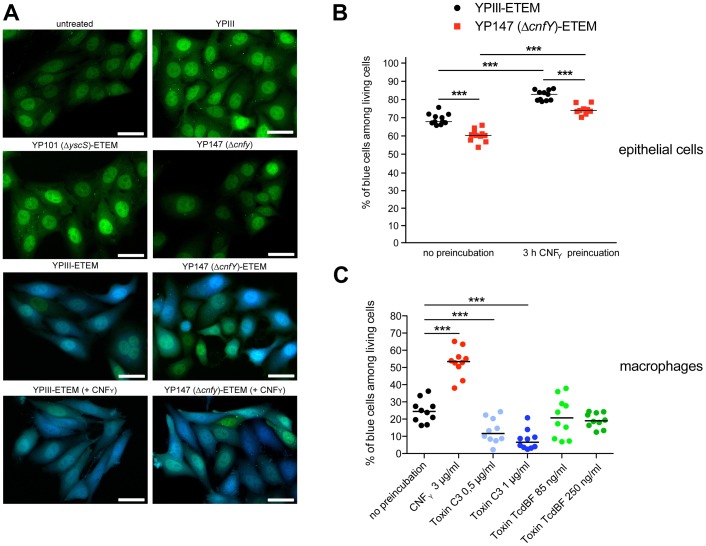
CNF_Y_-mediated increase of Yop delivery into epithelial cells and macrophages. (**A**) HEp-2 cells were untreated or treated with 25 nM purified CNF_Y_ toxin for 3 h prior to the infection with *Y. pseudotuberculosis* strains YPIII-ETEM (YP173), YP147 Δ*cnfY*-ETEM (YP217), and YP101 Δ*yscS*-ETEM (YP174). Cells were labeled with CCF4-AM and analyzed by fluorescence microscopy. Bar: 20 µm (**B**) HEp-2 cells were untreated or treated with 3 µg/ml (25 nM) purified CNF_Y_ toxin for 3 h prior to the infection with *Y. pseudotuberculosis* strains YPIII-ETEM (YP173) or YP147 Δ*cnfY*-ETEM (YP217) pregrown at 37°C. (**C**) Murine macrophages J774A.1 cells were untreated or treated with Rho GTPase modifying toxins CNF_Y_ (3 µg/ml), *C. botulinum* toxin C3 (0.5 µg/ml; 1 µg/ml) and *C. difficile* toxin TcdBF (85 ng/ml, 250 ng/ml) for 2 h. Subsequently, cell were infected with YPIII-ETEM pregrown at 37°C using a MOI of 10. Cells were labeled with CCF4-AM and percentage of blue HEp-2 cells and macrophages was determined. The graph represents data of two (**B**) or three (**C**) independent experiments with 5–6 wells per group. The asterisks indicate the significant difference in the quantity of translocated cells upon infection with the different strains or after toxin treatment based on a Mann-Whitney test with *** (P<0.001).

Since host actin polymerization by Rho activation plays a role in Yop translocation by *Y. pseudotuberculosis* [26] we also tested the influence of CNF_Y_ on Yop delivery. To do so, we generated *Y. pseudotuberculosis* strains expressing a YopE-β-lactamase reporter fusion [56], namely YP173 (YPIII-ETEM), YP174 (YP101Δ*sycS*-ETEM), and YP217 (YP147Δ*cnfY*-ETEM), and used these strains to infect host cells treated with the dye CCF4-AM. CCF4-AM consists of coumarin and fluorescein conjugated by a lactam ring and is modified by cellular esterases, whereby the dye becomes green fluorescent and is trapped inside the cell. If the β-lactam ring is cleaved by β-lactamase the dye changes its fluorescence from green to blue [57], [58]. The green to blue conversion allows identification of host cells in which the YopE-β-lactamase fusion protein has been successfully injected. We first used this fluorescence-based system to monitor translocation of the chimeric protein into HEp-2 cells, and determined the number of green and blue fluorescent cells by fluorescence microscopy and flow cytometry. Efficient translocation of YopE-β-lactamase into epithelial cells was observed upon infection with YP173 (YPIII-ETEM), but not with the secretion-deficient control strain YP174 (YP101Δ*yscS*-ETEM) (Fig. **8A,B**). YopE-β-lactamase translocation by the *cnfY*-deficient strain YP217 (YP147Δ*cnfY*-ETEM) was significantly reduced compared to YP173 (YPIII-ETEM), whereas preincubation of the host cells with CNF_Y_ increased translocation of the fusion protein (Fig. **8A, B**), indicating that CNF_Y_ enhances effector delivery. Since *Y. pseudotuberculosis* predominantly injects the Yops into professional phagocytes *in vivo* [59], we also tested CNF_Y_ influence on YopE-β-lactamase translocation into murine macrophages, and found that pretreatment with CNF_Y_ also boosts Yop delivery into these phagocytes (Fig. **8C**).

Stimulation of Rac1 through YadA and invasin-bound β_1_-integrins was shown to be essential for *Yersinia* uptake into epithelial cells [11], but neither internalization nor activation of Rac1 was required for Yop translocation by *Y. pseudotuberculosis* into HeLa cells [26]. This suggested that CNF_Y_-mediated stimulation of Yop delivery into macrophages might preferentially be caused by activation of RhoA. To validate this assumption, we pretreated macrophages with the *Clostridium botulinum* C3 toxin, an ADP-ribosylating protein that specifically inhibits RhoA, B and C, or with toxin B from variant *Clostridium difficile* serotype F strain 1470 (TcdBF), which specifically inhibits Rac but not RhoA/B/C [60], [61]. Treatment with the toxins induced actin cytoskeleton rearrangements and cell morphology changes, but had no effect on the viability of the macrophages and the number of associated bacteria (data not shown). As shown in Fig. **8C**, the RhoA/B/C inhibitor reduced the percentage of blue macrophages significantly, whereas the Rac inhibitor had no influence on YopE-β-lactamase translocation. These findings indicated that the CNF_Y_ toxin enhances Yop delivery into murine macrophages, and in particular activation of RhoA seems to play a role in the processes that stimulate Yop translocation into these professional phagocytes.

It has been reported that translocated effector YopE of *Y. pseudotuberculosis* YPIII is a GTPase-activating protein (GAP) for Rac1 and RhoA and this function appears important to regulate Yop translocation and modulate host defenses crucial for virulence [62]–[65]. This raised the question how YopE and CNF_Y_ contribute to RhoA-GTP and Rac1-GTP levels and Yop translocation. To address this, we analyzed RhoA and Rac1 activation and Yop translocation in the presence and absence of YopE in untreated or CNF_Y_-pretreated murine macrophages. As shown in Fig. **S7**, only low amounts of active Rac1 and RhoA could be detected in uninfected macrophages. Addition of the wild-type strain YPIII pregrown at 37°C to mimic the situation prior to host cell contact induced activation of RhoA and Rac1. Absence of YopE resulted in a small additional increase in RhoA-GTP, but had no or only a slight influence on Rac1-GTP levels. Furthermore, it had no or only a very small stimulatory effect on the translocation of YopD and YopH without or after pretreatment of the macrophages with CNF_Y_ (Fig. **S7**). This indicates that under these conditions intracellular YopE is unable to efficiently counteract CNF_Y_-mediated RhoA/Rac1 activation and reduce Yop translocation into murine macrophages.

We next analyzed whether the CNF_Y_ toxin affects Yop translocation into host cells in the original tissue environment. MLNs were harvested from uninfected mice and filtered to disrupt the tissue architecture and generate single-cell suspensions. Single cell suspensions were infected with a multiplicity of infection (MOI) of 10, incubated with CCF4-AM, and then analyzed by flow cytometry. As shown in Fig. **S8**, significantly higher numbers of blue cells with translocated YopE-β-lactamase were measured after infection with YPIII, indicating that Yop delivery into host cells can be enhanced by the toxin through activation of Rho GTPases.

### CNF_Y_ enhances Yop delivery into phagocytes during infection

It has been previously reported that *Y. pseudotuberculosis* selectively targets Yops to professional phagocytes in the PPs, MLNs and spleen during the oral route of infection [59]. To analyze whether the CNF_Y_ toxin also affects YopE-β-lactamase delivery in the course of an infection, we orally infected mice with 2×10^9^ bacteria YP173 and the isogenic *cnfY* mutant strain YP217. The T3SS-deficient *yscS* mutant, encoding the YopE-β-lactamase, and YPIII without the fusion were used as negative controls. At day three post infection mice were sacrificed, the PPs, MLNs, and spleen were harvested, and the translocation of Yops into various immune cell subsets was analyzed by flow cytometry (Fig. **S9**).

**Figure 9:**
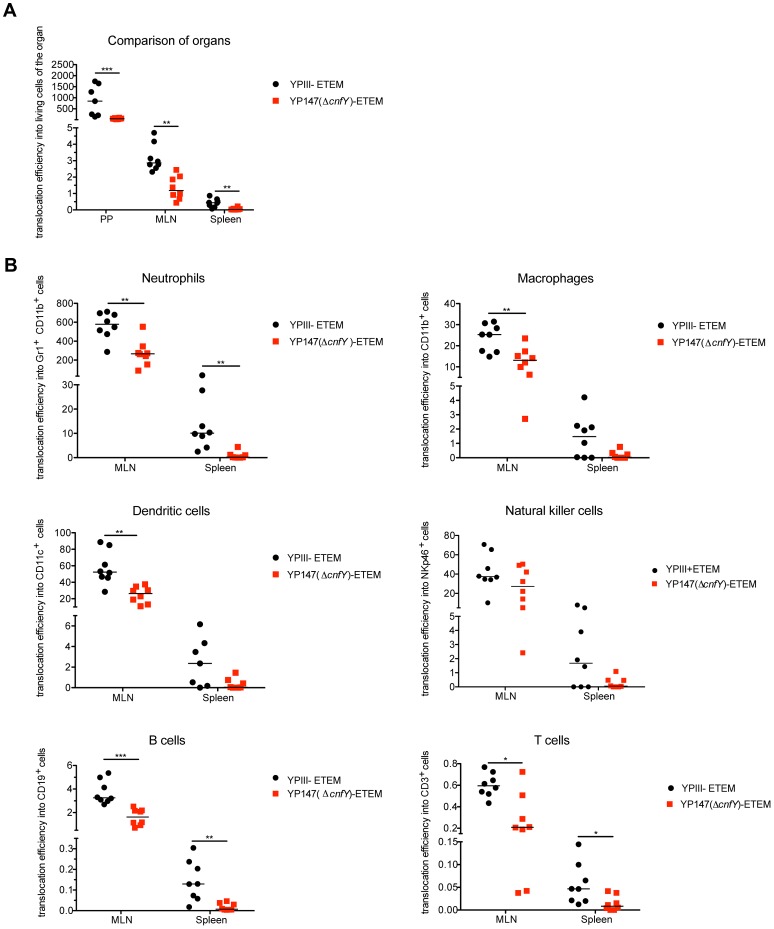
Absence of CNF_Y_ reduces Yop delivery into neutrophils, macrophages and DCs in PPs, MLNs and spleen during infection. (**A**) BALB/c mice were orally infected with 2×10^9^ cfu of YPIII-ETEM (YP173) and YP147 Δ*cnfY*-ETEM (YP217). YPIII and YP101 Δ*yscS*-ETEM (YP174) were used as negative controls. Day three post infection the MLNs, liver and spleen were isolated and filtred to generate single-cell suspensions. Cells were labeled with antibodies to the indicated surface markers for macrophages, DCs, neutrophils, NK cells, B and T cells and incubated with CCF4-AM. The percentage of blue cells was analyzed by flow cytometry (see also Fig. S10). Data of 8 mice of which the bacterial burden in the organs has been determined in parallel were normalized to the bacterial loads to determine Yop translocation efficiency. (**A**) Detection of green and blue cells by flow cytometry in PP, MLNs and spleen of mice infected with YP173 and YP217. The Yop translocation efficiency into all living cells is plotted. (**B**) The percentage of blue cells of the different analyzed cell types of the MLNs and the spleen were determined and normalized to the bacterial load in the tissues to determine Yop translocation efficiency. The asterisks indicate that Yop translocation efficiency in the different tissues differs significantly between YP147- and YPIII-infected mice based on a Mann-Whitney test with * (P<0.05), ** (P<0.01) and *** (P<0.001).

Following infection with the YopE-β-lactamase expressing wild-type strain 4.5% of all living cells within PPs were affected by Yop translocation. In contrast, only 1.5% of all living cells in the PPs contained the fusion protein after infection with the *cnfY*-deficient strain (Fig. **S10**). Yop translocation efficiency was still significantly reduced in tissues infected with the *cnfY*-deficient strain when the percentage of translocated blue cells was normalized to the bacterial load of the tissue/organ (Fig. **9**). This excludes that lower bacterial numbers account for this effect, but it also assumes that bacteria are infecting different cell types at the same MOI, which is unknown. Yop delivery was also significantly lower in the absence of the CNF_Y_ toxin in MLNs and spleen in which the total number of targeted cells was reduced compared to PPs (Fig. **9**, **S10**).

We further determined whether CNF_Y_-mediated stimulation of Yop translocation affected specific immune cells more frequently than others. Translocation of YopE-β-lactamase into each immune cell type was compared in MLNs and spleen from mice infected with YP173 (YPIII-ETEM) or the *cnfY* mutant derivative YP217 (YP147-ETEM) (Fig. **9B** **, S10**). In general, all immune cells analyzed were targeted by *Y. pseudotuberculosis*. However, Yop-injected neutrophils were significantly enriched in the MLNs and the spleen, indicating that this cell population is preferentially targeted in the tissues. In addition, DCs, NK cells and macrophages were well represented in the blue population, while B and T cells remained underrepresented (Fig. **9B** **, S10**). This is in full agreement with previous studies demonstrating that translocated YopH of *Y. pseudotuberculosis* strain IP2666 is enriched in neutrophils, macrophages and DCs in MLNs and spleen [**59**].

We further found that the apparent enhanced targeting to professional phagocytes, in particular neutrophils, macrophages and DCs and to a smaller extent also translocation into B and T cells was reduced in the absence of CNF_Y_ in the MLNs and spleen three days post infection (Fig. **9B** **, S10**). Yop translocation into NK cells was also somewhat reduced in the spleen. Since *Y. pseudotuberculosis* induces host cell death [66], which may be reflected in the strong reduction of professional phagocytes in the spleen (Fig. **6**), the actual amount of Yop translocation in this organ is probably underestimated. Taken together, these results demonstrate that the CNF_Y_ toxin plays a critical role during the infection, facilitating targeting of Yops to host immune cells, in particular professional phagocytes.

## Discussion

Many bacterial toxins and translocated effector proteins target Rho GTPases, which control crucial eukaryotic signal transduction pathways involved in the organization of the cell cytoskeleton, cell cycle progression, genetic information processing, and host defense processes to promote invasion, survival and replication of pathogens within their hosts [29], [67]. In this study we investigated the Rho-activating cytotoxic necrotizing factor CNF_Y_ of *Yersinia*. Although much progress has been made unravelling the molecular mechanism of this toxin, the functional consequences for host-pathogen interaction and pathogenesis were largely unknown. Using a murine model for gastrointestinal tract infections we provide evidence that this Rho-activating protein is crucial for virulence of the naturally toxin-expressing *Y. pseudotuberculosis* strain YPIII. The importance of CNF_Y_ for pathogenesis was first established by the analysis of the expression and the role of the toxin during the infection of mice. We show that *cnfY* is strongly expressed in all infected tissues during pathogenesis in mice, and is crucial for virulence, in particular for the dissemination of the bacteria into the MLNs, spleen and liver.

Histological analysis and immune cell composition of the infected tissues suggest that CNF_Y_ contributes significantly to the acute characteristics of the inflammatory response and host tissue damage during infection. Histo-pathologic evaluation underlines the finding that CNF_Y_ induces apoptosis, as focal necrosis was not seen in YP147-infected animals. Cell death leads to atrophy of the spleen in YPIII-infected mice. Moreover, a restriction of the inflammation to small foci could be observed in the intestine of YP147-infected animals, whereas the entire ileum was affected by a diffuse inflammation in YPIII-infected animals, explaining the shortening of the intestine. Hyperplasia of the white pulp seen in YP147-infected mice displays the immune response triggered by the infection. The infection is restricted to small foci in the intestine and is reversible, whereas the infection in YPIII infected animals is generalized and most probably leads to death by endotoxiemia. This inflammatory necrotizing phenotype is reminiscent of earlier studies analyzing the effect of CNF1 of *E. coli* using subcutaneous injections as well as animal models of urinary tract and prostatitis infection [31], [68], [69].

Infections of the gastrointestinal tract by enteropathogenic *Yersiniae* lead to a biphasic inflammatory process in which bacterial adhesion and transmigration through the intestinal epithelial layer triggers an initial antibacterial defense response with little inflammation, e.g. expression of IL-8 by epithelial cells, which is followed by an acute infiltration and activation of neutrophils, cytokine production and tissue necrosis [70]. First recognition of *Y. pseudotuberculosis* occurs through contact of the bacterial LPS with TLR4 on naïve host macrophages and this leads to proinflammatory cytokine production through activation of MAPK and NF-_k_B. However, translocation of YopJ inhibits activation of MAPK and NF-κB and induces an apoptotic signaling pathway including activation of initiator caspase-8, and the executioner caspase-3, -7, and -9 [70], [71]. Apoptotic macrophages are eliminated and this process also triggers production of anti-inflammatory cytokines such as IL-10 and TGF-β [72], [73]. However, induction of apoptosis is probably not always fully immunologically silent, e.g. phagocytosis of apoptotic cells by other phagocytes, can prime other immune responses such as activation of CD8^+^ T cells [71]. During the course of the infection, the number of activated macrophages increases whereas the number of naïve macrophages declines. In activated macrophages *Yersinia* causes cell death by inflammatory pyroptosis. This occurs through activation of a multiprotein complex, called the inflammasome, which forms a platform for the autoprocessing and activation of the cysteine protease caspase-1. Activation of caspase-1 results in the secretion of the inflammatory cytokines such as IL-1α, IL1-β, and IL-18, and triggers cell death [66]. LPS, the T3SS and the translocated YopJ protein of *Yersinia* were shown to induce caspase-1 activation and pyroptosis [71], [74].

Induction of pyroptosis (inflammatory death) in activated macrophages corresponds to later stages of the infection with *Y. pseudotuberculosis*, where inflammation and necrosis is evident from histopathology. Based on our results it is very likely that CNF_Y_ supports *Yersinia*-induced pyroptosis of activated phagocytes in the spleen during later stages of the infection. CNF_Y_ was shown to manipulate the number of immune cells and induce inflammatory responses. The number of macrophages, monocytes and neutrophils decreased strongly (4- to 15-fold) in all lymphoid organs three days post infection. In contrast, infection with the *cnfY* mutant resulted in no reduction, but rather in an increase of phagocytes when compared to uninfected control mice, and the overall inflammation of the infected tissues was considerably reduced. Similar to CNF1 of *E. coli* [34], it is possible that CNF_Y_ is transported by OMVs, which act as long-range toxin delivery vectors, and is then able to reduce chemotaxis and influx of professional phagocytes by constitutive Rho GTPase activation. We further demonstrate that CNF_Y_ enhances Yop delivery into phagocytes during infection. This strongly suggests that increased translocation of YopJ could stimulate cell death in the spleen. Consistent with this assumption, YopJ and CNF_Y_ promote systemic dissemination following oral infection. Work by Monack *et al.* [75] showed that a *yopJ* mutant is deficient for spread from the PPs to other lymphoid tissues (MLNs, spleen), similar to the *cnfY* mutant investigated in this study. Moreover, wild-type *Yersinia* induce apoptosis of macrophages from infected spleens [75], implying that mainly YopJ is used to eliminate immune cells in the spleen to dampen the immune response against *Yersinia* during infection. YopJ was also shown to subvert the NOD2/RICK/TAK1 pathway, activate caspase-1 and induce IL-1β secretion within PPs, which is associated with increased barrier permeability [76]. This suggests that CNF_Y_ action also enhances YopJ-dependent intestinal barrier disruption and promotes the dissemination of *Yersinia* by exploiting the mucosal inflammatory response. In addition, CNF_Y_ seems to contribute to depletion of NK cells in the spleen. It was observed that *Y. pestis* but not an isogenic *yopM* deficient mutant caused a significant global decrease in NK cell numbers [21], indicating that NK cell depletion is enhanced by CNF_Y_-mediated activation of YopM translocation.

Here, we observed that RhoA, Rac1 and Cdc42 are activated in CNF_Y_-intoxicated macrophages, which is reflected by the high content of actin cables/stress fibres, the formation of lamellipodia and filopodia, pronounced cell spreading and inhibited cytokinesis. Previous work demonstrated that CNF_Y_ predominantly activates RhoA in epithelial cells [27], [28]. However, a very recently published study also reported CNF_Y_-mediated activation of Rac1 and Cdc42 in HeLa cells [77]. Use of different toxin concentrations, incubation times and cell types (human epithelial cells versus murine macrophages) in which the CNF toxins may display a different selectivity and different efficiencies of cell toxifications are likely to account for these variations. In fact, CNF_Y_-mediated Rho GTPase activation pattern varies during intoxication whereby RhoA activation is generally more pronounced than Rac1 and Cdc42 2–3 h after toxin addition (J. Schweer, unpublished results, [77]). This suggests that at very early time points and/or under low toxin concentrations predominantly RhoA might be activated. A previous report demonstrates that *Y. pseudotuberculosis* selectively modulates RhoA activity (e.g. by signals triggered by the YopB/D translocon and/or from engagement of β_1_-integrin receptors) to induce cellular changes that control T3SS pore formation and effector translocation [26]. Here, we strengthen this observation, as CNF_Y_-mediated stimulation of Yop delivery of *Y. pseudotuberculosis* was sensitive to the Rho inhibitor C3-transferase of *C. botulinum*, but insensitive to Rac1 inhibition by TcdBF toxin of *C. difficile*. In contrast, new experiments addressing the influence of CNF_Y_ on Yop translocation of *Y. enterocolitica* demonstrated that CNF_Y_ also stimulates effector delivery by this pathogen, although this process seemed entirely dependent on Rac and not on Rho GTPases [77]. Different YadA/InvA-promoted signalling events, differences in Yop protein abundance (e.g. RhoA-inactivating YopT is absent in YPIII) and differences in the regulation of Yop delivery by Rho GTPases between the different species may be responsible for this discrepancy.

Some effector proteins, in particular YopE, were shown to inhibit Yop delivery by inactivation of RhoA and Rac1 most likely as part of an intra-cellular control mechanism which measures and adapts the amount of protein translocated by *Yersinia* during infection. This is reflected by elevated levels of Yop effector translocation into epithelial cells by *yopE*-deficient strains [11], [63]–[65], [78]. Our analysis demonstrated that absence of YopE caused no or only a small increase in Rac1/RhoA activation and Yop translocation during infection of murine macrophages with *Y. pseudotuberculosis* YPIII with or without treatment with CNF_Y_. This indicates that intracellular YopE is not able to counteract CNF_Y_ in these phagocytes. Recently published work showing that none of the Rho inhibiting effectors (YopE, YopT and YopO) could reduce the effect of CNF_Y_ on Yop translocation by *Y. enterocolitica* into human epithelial cells supports this observation [77]. However, we cannot exclude the possibility that other conditions (e.g. conditions which enhance (i) deamidation and subsequent ubiquitin-dependent degradation of the modified Rho GTPases or (ii) YopE translocation and activity) allow counterregulation.

Very recently, it has also been reported that NOD1, a pattern recognition receptor that senses cytosolic microbial products similar to NOD2, monitors the activation state of all three Rho GTPases. Activation of Rho GTPases triggered the NOD1 signalling cascade with consequent RIP2-mediated induction of NF-κB-dependent inflammatory responses [79]. NOD1 activation was triggered by activation of Rac1 and Cdc42 by the *Salmonella* effector SopE. In line with this, all three Rho GTPases, Rac1, RhoA and Cdc42 were previously shown to activate the NF-κB pathway [80] and particular Rac1 has been reported to contribute to NF-κB activation by CNF1 of *E. coli* by clustering the NF-κB inhibitor IκBa and components of the IκBα E3-ubiquitin ligase into membrane ruffles [79].

**Figure 10:**
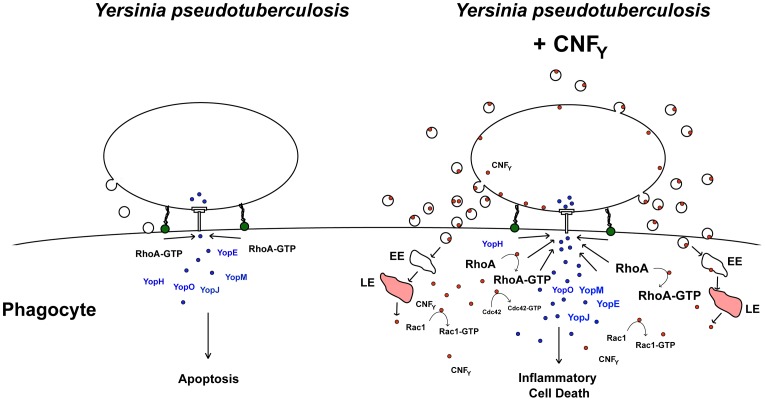
Model of CNF_Y_ toxin function. Adhesin-mediated interaction of *Y. pseudotuberculosis* with macrophages induces activation of RhoA which induces T3SS-promoted Yop delivery into the phagocyte. Yop translocation leads to blockage of phagocytosis and YopJ-mediated apoptotic cell death. In CNF_Y_-producing *Y. pseudotuberculosis*, prior or parallel to the interaction of the bacteria with phagocytes, the CNF_Y_ toxin is secreted via outer membrane vesicles. Most likely CNF_Y_-containing vesicles are internalized through receptor-mediated endocytosis, transferred via early endosomes (EE) to acidic compartments (late endosomes, LE) where the toxin is translocated across the bilayer into the cytosol by a low pH-dependent mechanism. Liberation of CNF_Y_ leads to activation of small Rho GTPases, in particular RhoA, and this amplifies Yop delivery and cell death. Rho GTPase activation was shown to promote pore formation of the T3SS, which appear to activate caspase-1 [26], [74]. Furthermore, enhanced cell death can lead to an accelerated proinflammatory immune response known to raise the level of activated macrophages in which *Yersinia* triggers activation of caspase-1 [66]. Induction of caspase-1 leads to secretion of inflammatory cytokines and triggers cell death by inflammatory pyroptosis. In parallel, uncontrolled proliferation of the bacteria will lead to higher toxin concentrations, which will further enhance activation of Rac1, Cdc42 and RhoA and potentiate inflammatory pyroptosis.

Based on our current knowledge we envision a model in which the CNF_Y_ toxin exerts its function in a multi-step process (Fig. **10**). The first step corresponds to the uptake of the CNF_Y_ toxin by infiltrating innate immune cells (e.g. neutrophils, macrophages, DCs) in the early phase of the infection process. This triggers activation of the Rho GTPases, in particular RhoA, in the phagocytes. Induced actin polymerization resulting from Rho GTPase activation enhances Yop delivery into host cells to counteract innate and adaptive immune responses. As a consequence, invading immune cells are inhibited and undergo apoptosis leading to uncontrolled proliferation of the pathogens. Higher CNF_Y_ toxin concentrations by replicating pathogens potentiate activation of RhoA, Rac1 and Cdc42 which triggers inflammatory responses e.g. via the NOD1-RIP2 signalling cascade. In addition, interaction of *Yersinia* with increased numbers of activated macrophages causes cell death by inflammatory pyroptosis leading to strong inflammation and necrosis of the organs during later phases of the infection process. In summary, our data identify CNF_Y_ as an important Rho GTPase-activating toxin which is instrumental for *Yersinia* to amplify crucial virulence factor functions which determines the success of the infection and the severity of the associated disease.

## Material and Methods

### Ethics statement

All animal work was performed in strict accordance with the German Recommendations of the Society for Laboratory Animal Science (GV-SOLAS) and the European Health Recommendations of the Federation of Laboratory Animal Science Associations (FELASA). The animal protocol was approved by the Niedersächsisches Landesamt für Verbraucherschutz und Lebensmittelsicherheit: animal licensing committee permission no. 33.9.42502-04-055/09. Animals were handled with appropriate care and welfare, and all efforts were made to minimize suffering.

### Bacterial strains, cell culture, media and growth conditions

The strains used in this study are listed in **Table 1**. Overnight cultures of *E. coli* were routinely grown at 37°C, *Yersinia* strains were grown at 25°C or 37°C in LB (Luria-Bertani) broth. The antibiotics used for bacterial selection were as follows: carbenicillin 100 µg/ml, chloramphenicol 30 µg/ml, kanamycin 50 µg/ml, and gentamicin 50 µg/ml. For infection experiments, bacteria were grown at 25°C or 37°C, washed and diluted in PBS prior to infection. For invasion assays and mouse infections, bacteria were grown to stationary phase, washed and resuspended in PBS. For the *in vitro* Yop delivery assay, bacteria were grown in LB medium at 37°C.

**Table 1:** Bacterial strains and plasmids.

Strains, Plasmids	Description	Source and reference
**Bacterial strains**		
*E. coli* K-12		
DH101β	F^−^ *endA1 recA1 galE15 galK16 nupG rpsL ΔlacX74*	Invitrogen
	Φ80*lac*ZΔM15 *araD139* Δ(*ara,leu*)7697 *mcrA*	
	Δ(*mrr-hsdRMS-mcrBC*) λ^−^	
BL21 λDE3	F^−^ *ompT gal dcm lon hsdSB*(r_B_^−^ m_B_^−^) *gal* λDE3	[86]
CC118 λ*pir*	F^−^ Δ(*ara-leu*)7697 Δ(*lacZ*)74 Δ(*phoA*)20 *araD139*	[87]
	*galE galK thi rpsE rpoB arfE* ^am^ *recA1*	
S17-1 λpir	Tp^r^ Sm^r^ *recA*, *thi*, *pro*, *hsdR* ^−^ *M* ^+^ RP4:2-Tc:Mu:Km Tn7	[88]
	λpir	
*Y. pseudotuberculosis*		
YPIII	pIB1, wild-type	[89]
YP56	YPIII Δ*phoPQ*, Kan^R^	this study
YP101	YPIII, Δ*yscS*, Kan^R^	Rebekka Steinmann
YP147	pIB1, Δ*cnfY*, Kan^R^	this study
YP149	YPIII *phoPQ* ^IP32953^	this study
YP173	YPIII ETEM, amino acids 1 to 100 of YopE+TEM1	this study
YP174	YPIII Δ*yscS* ETEM,	this study
	amino acids 1 to 100 of YopE+TEM1	
YP188	YP149 Δ*cnfY*, Kan^R^	this study
YP217	YP147 ETEM, amino acids 1 to 100 of YopE+TEM1	this study
YP275	pIB1, Δ*yopE*, Kan^R^	this study
IP32953	wild-type, pYV	[90]
**Plasmids**		
pACYC177	cloning vector, p15, Ap^R^, Kan^R^	[91]
pAKH3	pGP704, *sacB^+^*, Amp^R^	[83]
pDM4	R6K derivative, *sacB^+^*, Cm^R^	[92]
pET28a	T7 promoter based expression vector, Kan^R^	Novagen
pFU54	promoterless *luxCDABE*, pSC101* ori, Amp^R^	[93]
pFU58	promoterless *gfpmut3.1*, pSC101* ori, Amp^R^	[93]
pFU68	promoterless *lacZ*, pSC101* ori, Amp^R^	[93]
pFU166	*gapA-luxCDABE*, colE1 ori, Amp^R^	[93]
pFU228	*gapA-dsRed2*, colE1 ori, Cm^R^	[93]
pFU234	*ifp* ^+^, pSC101* ori, Kan^R^	[94]
pKOBEG-*sacB*	recombination vector, *sacB^+^*, Cm^R^	[95]
pSR47s-E-TEM2	YopE-TEM1; Kan^R^	[56]
pJNS01	pET28a, *cnfY*, Kan^R^	this study
pJNS02	P*cnfY-luxCDABE*, pSC101* ori, Amp^R^	this study
pJNS03	P*cnfY-gfpmut3.1*, pSC101* ori, Amp^R^	this study
pJNS04	P*cnfY-lacZ*, pSC101* ori, Amp^R^	this study
pJNS05	pAKH3, *cnfY*::Kan^R^, *sacB^+^*, Amp^R^	this study
pJNS09	pFU234, Amp^R^	this study
pJNS10	pJNS09, *cnfY* ^+^, pSC101* ori, Amp^R^	this study
pJNS11	pJNS09, pSC101* ori, Amp^R^	this study
pJNS13	pAKH3, *yopE*::Kan^R^, *sacB^+^*, Amp^R^	this study
pVP1	pDM4, *phoPQ* ^IP32953^	this study
**Primers**		
360	5′-GGTGATTTTGAACTTTTGCTTTG-3′	
361	5′-CCAGTGTTACAACCAATTAACC-3′	
538	5′-GTCGTGGGTGCCAGCCG-3′	
539	5′-GCAAAGCAAAAGTTCAAAATCACCCCAATCCTTGATAAAACGTTAACCGGATAACAGGATATTATCCGGTAAG-3′	
540	5′-GGTTAATTGGTTGTAACACTGGGCCAGTCAGCCCCTGATACTGATGGCGGGTATCCGTTATAATCTCAGG-3′	
541	5′-CCAGCGGCGACGGCCTG-3′	
I661	5′-GTGTAGGCTGGAGCTGCTTC-3′	
I662	5′-CATATGAATATCCTCCTTAGTTCC-3′	
II794	5′-GGGGCTAGCATGAAAAATCAATGGCAA-3′	
II795	5′-GGGCTCGAGTTAAAAGTCTTTTTGTAA-3′	
II896	5′-GGGGGGGATCCTATTGACAAACAAAATGAAGCAAGATAG-3′	
II898	5′-GGGGGGGATCCCAGAATATGGTGAGCATAGGGAATGA-3′	
III710	5′-CCGGGGAGCTCGACAAACAAAATGAAGCAAGATAGTTTTACATG-3′	
III712	5′-CCGGGGAGCTCATTTTCTGGCGGGGTGTGACCA-3′	
III714	5′-GGAACTAAGGAGGATATTCATATGTAATGTTTTACAAAAAGACTTTTAAATCTTAAGTCTC-3′	
III715	5′-GAAGCAGCTCCAGCCTACACAAACACTCCTTTTATTGACGATGCACAATG-3′	
III926	5′-GCGCGAGCTCCCAGCGGCGACGGCCTG-3′	
III927	5′-GCGCCTCGAGGTCGTGGGTGCCAGC-3′	
IV16	5′-GGGGGGCGGCCGCTTAAAAGTCTTTTTGTAAAAC-3′	
V553	5′-CCGGGGAGCTCAGATGGGTTTTAATATATCTTGACTGAAT-3′	
V554	5′- GAAGCAGCTCCAGCCTACACTTCTCCTACGCTGCTAGATCC-3′	
V555	5′-GGAACTAAGGAGGATATTCATATGTATTGATAAAAACAAGGGGATAGTGTT-3′	
V556	5′- CCGGGGAGCTCAATGTACCTGTGAGCCATCG-3′	

Human HEp-2 cells were cultured in RPMI 1640 media with an alternative to L-glutamine with increased stability (Invitrogen) supplemented with 7.5% newborn calf serum (Sigma Aldrich). Murine J774A.1 macrophages were cultured in the same medium supplemented with 5% fetal calf serum (PAA). All cell lines were cultivated at 37°C in the presence of 5% CO_2_.

### DNA manipulations and construction of plasmids

All DNA manipulations, PCR, restriction digestions, ligations and transformations were performed using standard techniques as described previously [81], [82]. Plasmids used in this study are listed in **Table 1**. Plasmid pJNS01 (*cnfY*_his6_) was constructed by amplification of *cnfY* (*ypk_2615*) from genomic DNA of *Y. pseudotuberculosis* YPIII with primers II795/II795 and integrated into the *Xho*I/*Nhe*I sites of pET28a (Novagen).

For construction of the *luxCDABE*, *gfp*mut3.1 and *lacZ* reporter gene fusions encoded on plasmids pJNS2-4, the promoter region of *cnfY* (primer pair II896/II898) was amplified and ligated into the *Bam*HI/*Sal*I sites of pFU54, pFU58 and pFU68.

Plasmid pJNS10, containing the *cnfY* promotor region with the *cnfY* gene, was constructed by the insertion of a PCR fragment amplified with primers II896 and IV16 from chromosomal DNA of *Y. pseudotuberculosis* YPIII into the *Bam*HI*/Not*I sites of pFU234. All clones were confirmed by sequencing and restriction.

### Construction of mutant strains

For generation of the mutant strain YP147, a *cnfY*::Kan^R^ mutation encoded on plasmid pJNS05 was constructed. To do so, the kanamycin resistence gene was amplified using the *kan* primers (I661/I662) and plasmid pACYC177 as template. Next, *Y. pseudotuberculosis* YPIII genomic DNA was used as a template to amplify 500-bp regions flanking the target gene *cnfY*. The upstream fragment was amplified with the primer pair III710/715 of which the reverse primer contained 20 nt at the 5′-end which are homologous to the start of the kanamycin resistance gene. The downstream fragment was amplified with primer pair III712/III714 of which the forward primer contained additional 20 nt at the 3′-end which are homologous to the end of the kanamycin resistance gene. A PCR reaction was performed with the forward primer and the reverse primer using the upstream and downstream PCR products of the target gene and the kanamycin gene fragment as templates and cloned into pAKH3. The resulting plasmid pJNS05 was integrated into the *cnfY* locus of YPIII via conjugation as described [83]. Chromosomal integration of the fragments was selected by plating on LB supplemented with kanamycin. Excision of the plasmid including the defective *cnfY* allele of YPIII was obtained by plating of the strain on 10% sucrose and generated strain YP147 was analyzed by PCR and DNA sequencing. For the construction of strain YP56 (Δ*phoPQ*), a *phoPQ*::Kan^R^ PCR fragment was generated. For this purpose, the kanamycin resistance cassette was created using the primers 360/361 and pACYC177 as template. The primers contain homology regions (20 nt) to the upstream or downstream region of the *phoPQ* gene. A fragment including sequences of the *phoPQ* upstream region was generated by PCR using the primers 538/539, a fragment including sequences of the *phoPQ* downstream region was amplified using the primers 540/541. Primer 539 and 540 contain 20 nt of the kanamycin resistance cassette. A PCR fragment consisting of these three fragments was amplified using the primers 538/541. The product was transformed into *Y. pseudotuberculosis* YPIII pKOBEG-*sacB* and a *phoPQ*::Kan^R^ mutant (YP56) was generated and selected for as described [84]. For the construction of YP149, the *phoPQ* gene of strain IP32953 was amplified by PCR with primer III926 and III927, creating *Sac*I and *Xho*I restriction sites. The fragment was cloned into plasmid pDM4, generating vector pVP1. Integration of the plasmid was obtained through conjugation of strain S17λpir pVP1 with the YP56 as described [83]. Excision of the plasmid including the defective *phoPQ* allele was obtained by plating of the strain on 10% sucrose. PCR and DNA sequencing proved presence of the intact *phoPQ* allele. To generate strain YP188 the *cnfY* gene was destroyed as described above for YP147 (Δ*cnfY*). Strains YP173, YP174 and YP217 were constructed by chromosomal integration of the YopE-β-lactamase (ETEM) fusion plasmid pSR47s-E-TEM1 into the *yopE* locus. Integration was obtained through conjugation of *E. coli* K-12 strain S17λpir pSR47s-E-TEM1 with the *Y. pseudotuberculosis* strains YPIII, YP101 (Δ*yscS*) and YP147 (Δ*cnfY*) as described [83]. For generation of the mutant strain YP275, a *yopE*::Kan^R^ mutation encoded on plasmid pJNS13 was constructed as described above for YP147. Primer V553/V554 and V555/V556 were used to amplify 500-bp regions flanking the target gene *yopE*. Subsequently, plasmid pJNS13 was integrated into the *yopE* locus of YPIII via conjugation as described [83].

### β-galactosidase assays

β-galactosidase activity was determined of three independent cultures of bacteria harboring the *cnfY-lacZ* fusion as described [83]. The activities were calculated as follows: β-galactosidase activity OD_420_ • 6.75 OD_600_^−1^ • Δt (min)^−1^ • vol (ml)^−1^.

### Purification of CNF_Y_-His_6_

For overexpression of CNF_Y_ *E. coli* strain BL21λDE3 was transformed with the *cnfY* expression plasmid pJNS01 and grown at 37°C in LB medium to an OD_600_ of 0,6. Subsequently, P*_lac_*-driven expression was induced upon addition of 250 µM IPTG and grown at 17°C overnight. CNF_Y_-His_6_ production was tested by westernblot analysis using an antibody directed against the His-tag (Qiagen). For purification of CNF_Y_, cells were harvested, resuspended in 50 mM NaH_2_PO_4_, pH 8.0, 300 mM NaCl, 10 mM imidazole and lysed with a French press (120.000 psi). The soluble CNF_Y_-His_6_ extract was separated from insoluble cell material by centrifugation at 25.000 g. The CNF_Y_-His_6_ protein was then purified by affinity chromatography on Ni-NTA agarose (Qiagen). The column was washed with three column volumes of 50 mM NaH_2_PO_4_, pH 8.0, 300 mM NaCl, 20 mM imidazole and eluted with 50 mM NaH_2_PO_4_, pH 8.0, 300 mM NaCl containing 250 mM imidazole.

### Visualization of the actin cytoskeleton

In order to study the influence of the recombinant CNF_Y_ protein on actin cytoskeleton rearrangements, 1×10^5^ J774A.1 cells were incubated with purified CNF_Y_ toxin (10 nM) or PBS for 24 h. Subsequently, cells were fixed with 4% paraformaldehyde (in PBS) for 10 min at room temperature, washed with PBS and permealized with 0,1% Triton X-100 in PBS for 5 min. The actin cytoskeleton was stained with Phalloidin-FITC (0,5 µg/ml PBS; Invitrogen) for 15 min at room temperature. Cells were washed in PBS, and the nuclei were stained with DAPI (1 µg/ml in TBST) for 5 min at room temperature. Cells were visualized using a fluorescence microscope (Axiovert II with Axiocam HR, Zeiss, Germany) and the AxioVision program (Zeiss, Germany).

### Rho GTPase activation assay

Activation of RhoA was tested using the Rho activation assay kit 17-294 (Millipore, Billerica, MA, USA) and activation of Rac1 and Cdc42 was determined with the Rho/Rac/Cdc42 Activation Assay Combo Kit (Cell Biolabs, San Diego, CA, USA). Approximately 1×10^6^ cells of the macrophage cell line J774A.1 were starved for at least 20 h in RPMI 1640 without FCS, and incubated with PBS or 25 nM (3 µg/ml) recombinant CNF_Y_ for 2–3 h. To test the influence of YopE on Rac1 and RhoA activation, macrophages were subsequently infected for 20 min with wild-type strain YPIII or the isogenic *yopE* mutant YP275 with an MOI of 100. Cells were lysed and activation of small Rho GTPases was tested and visualized by western blotting according to the manufacturer's protocol.

### Yop delivery assay

The Yop delivery assay was performed as described previously [85]. 5×10^4^ (for fluorescence microscopy) or 1×10^6^ (for flow cytometry) HEp-2 or J774A.1 cells were incubated with recombinant CNF_Y_ (25 nM/3 µg/ml), exoenzyme C3 transferase from *C. botulinum* (CT04, Cytoskeleton. Inc) (0.5 µg/ml, 1 µg/ml), *C. difficile* toxin TcdBF (85 ng/ml, 250 ng/ml) [60], [61], or the same amount of PBS before the cells were infected with bacteria with a MOI of 10. After 1 h cells were washed and dyed with CCF4-AM according to the manufacturer's protocol using the LiveBLAzer-FRET B/G Loading Kit from Life Technologies. Yop translocation was visualized by a fluorescence microscope (Axiovert II with Axiocam HR, Zeiss, Germany) using the AxioVision program (Zeiss, Germany) or detected with an LSR Fortessa cell analyzer (BD Bioscience). Acquired data of flow cytometry were then analyzed with FlowJo software (Treestar).

To compare Yop translocation of YPIII and YP275 (Δ*yopE*), both strains were pregrown at 37°C and added with an MOI of 100 to approximately 1×10^6^ cells of murine macrophages incubated with PBS or 25 nM recombinant CNF_Y_ for 3 h. One hour post infection, cells were washed with PBS, resuspended in SDS sample buffer and separated on 12% SDS polyacrylamide gels. Proteins were blotted onto a membrane and intracellular Yops were visualized with an antiserum directed against all secreted Yops (α-Yop).

For the *in vitro* analysis of Yop delivery into primary cells, mesenteric lymph nodes from uninfected 6- to 8-week-old BALB/c mice were removed. To generate single-cell suspensions the cells were pressed through a 70 µm cell strainer. Harvested cells were counted using an Accuri C6 flow cytometer (BD Bioscience). Bacteria were cultured overnight at 25°C in LB medium, inoculated 1∶20 in fresh LB and grown for 3 h at 37°C prior to infection. Cells were infected for 1 h at 37°C with the wild-type (YP173), the *cnfY* (YP217) or the *yscS* (YP174) mutant encoding the YopE-β-lactamase fusion – ETEM with an MOI of 10. Infected cells were washed twice with RPMI 1640 medium supplemented with 20 mM HEPES (pH 7.0), 0.4% BSA and 50 µg/ml gentamicin to kill bacteria. Subsequently, 2×10^6^ cells were labeled with LiveBLAzer-FRET B/G Loading Kit from Life Technologies. After staining for 1 h at room temperature, cells were prepared for flow cytometry and analyzed as described above.

### Mouse infection

BALB/c female mice aged between 6- and 8-week-old were purchased from Janvier (Saint Berthevin Cedex, France) and housed under specific pathogen-free conditions according to FELASA recommendations in the animal facility of the Helmholtz Centre for Infection Research, Braunschweig. For the survival assays, mice were infected orally with approximately 2×10^9^ bacteria of each strain. The infected mice were monitored for 14 days every day to determine the survival rate, the body weight and health status.

Bacteria used for organ burden experiments were grown over night in LB medium at 25°C, washed and resuspended in PBS. Groups of 7–10 animals were orally infected with approximately 2×10^8^ bacteria of *Y. pseudotuberculosis* strains YPIII and YP147 (Δ*cnfY*) using a gavage needle. At specific time points after infection, mice were euthanized by CO_2_ asphyxiation. PPs, small intestine, caecum, MLNs, liver and spleen were isolated. The ileum was rinsed with sterile PBS and incubated with 100 µg/ml gentamicin in order to kill bacteria on the luminal surface. After 30 min, gentamicin was removed by washing with PBS. Subsequently, all organs were weighted and homogenized in PBS at 30.000 rpm for 30 sec using a Polytron PT 2100 homogenizer (Kinematica, Switzerland). To determine the bacterial load of the organs serial dilutions of the homogenates were plated on LB plates with and without antibiotics. The colony forming units (cfu) were counted and are given as cfu per g organ/tissue.

### Yop delivery assay during mouse infection

BALB/c mice were infected intragastrically with 2×10^9^ bacteria of strain YPIII-ETEM (YP173) and the isogenic *cnfY* mutant YP147-ETEM (YP217), wild-type YPIII and YP101-ETEM (YP174). Infection was allowed to proceed for three days. Subsequently, the infected lymphatic tissues (PPs, MLNs, and spleen) were isolated and single cell suspensions were generated in PBS by pressing the cells through a cell strainer (70 µm, Falcon). To eliminate erythrocytes, spleen cells were incubated for 3 min in lysis buffer (7.8 mM NH_4_Cl, 10 mM KHCO_3_, 100 µM EDTA). All cells were resuspended in PBS containing 0.2% BSA and total cell number was determined using an Accuri C6 flow cytometer (BD Bioscience). For flow cytometry analysis 1×10^6^ cells were transferred per tube and FcγR was blocked using CD16/CD32 (BioXCell; anti-mouse CD16/CD32) antibody for 15 min at 4°C. Immune cells were first stained for 15 min at 4°C using a biotin-conjugated antibody against CD19. Subsequently, other cellular surface marker for innate immune cells or T cell panel were stained for 20 min at 4°C in FACS buffer (PBS+0.2% BSA) using the following antibodies: SA-PerCP-Cy5.5, CD11c-APC, CD11c-PE-Cy7, Gr1-A750, CD3-PE, CD4-APC-Cy7, CD3-PE-Cy7, NKp46-PE and CD25-APC. Samples were washed twice in FACS buffer and labeled with 1 µg/ml CCF4-AM using the LiveBLAzer-FRET B/G Loading Kit (Life Technologies) for 1 hour at 20°C in the presence of 1.5 mM probenecid (Sigma) and 50 µg/ml gentamicin. Cell subsets were defined as following: B cells (CD19^+^ CD3^−^), T cells (CD19^−^ CD3^+^), NK cells (CD19^−^ CD3^−^ NKp46^+^), neutrophils (CD19^−^ CD3^−^ CD49b^−^ Ly6G^+^ CD11b^+^), macrophages/monocytes (CD19^−^ CD3^−^ CD49b^−^ Ly6G^−^ CD11b^+^), and DCs (CD19^−^ CD3^−^ CD49b^−^ Ly-6G^−^ B220^−^ F4/80^−^ CD11c^+^). Cells were analyzed in a LSR Fortessa cell analyzer (BD Bioscience). Acquired data were analyzed with FlowJo software (Treestar). Cells from tissues that were not treated with CFF4-AM and/or antibodies were used as negative controls.

### CNF_Y_ influence in the host immune response

To characterize the host immune response induced upon infection with the wild-type strain YPIII or the isogenic *cnfY* mutant strain YP147, mice were orally infected with approximately 2×10^8^ bacteria of *Y. pseudotuberculosis* strains YPIII or YP147 (Δ *cnfY*). Three and six days after infection, PPs, MLNs and spleen were isolated. Single cell suspensions were obtained by mechanical disruption of the organs through a cell strainer. To eliminate erythrocytes, spleen cells were also incubated for 3 min in erythrolysis buffer (7.8 mM NH_4_Cl, 10 mM KHCO_3_, 100 µM EDTA). All cells were resuspended in FACS buffer and total cell number was determined using an Accuri C6 flow cytometer (BD Bioscience). Amounts of 1–2×10^6^ cells were transferred per tube. To exclude dead cells from the analysis live/dead staining (Invitrogen; Live/dead fixable blue dead cell stain kit, UV excitation) was performed for 30 min. FcγR and IgG were blocked by 15 min incubation with CD16/CD32 (BioXCell; anti-mouse CD16/CD32) and ratIgG (Jackson ImmunoResearch; ChromPure Rat IgG, whole molecule) antibodies.

Cellular surface markers for either lymphoid or myeloid panel were stained for 15 min at 4°C in PBS-BSA (0.2%) using the following antibodies: CD3-APC, CD4-PerCP-Cy5.5, CD8-eFluor450, CD335-PerCP-Cy5.5, CD11b-PacificBlue, CD19-Biotin, CD45R-PerCP-Cy5.5, F4/80-PE and CD11c-APCeFluor780 from BD Bioscience, and CD19-FITC, CD49b-Biotin, and Ly-6C-APC from BioLegend. All antibodies were titrated for optimal staining conditions. Biotin-conjugated antibodies were incubated with streptavidin for 15 min at 4°C. After staining cells were fixed with the Foxp3 staining buffer set from eBioscience. Cells were then washed twice and resuspended in 200 µl FACS buffer. Samples were loaded into an LSR Fortessa cell analyzer (BD Bioscience). Acquired data were analyzed with FlowJo software (Treestar).

### *In vivo* expression of CNF_Y_

YPIII harboring a P*_cnfY_*::*gfpmut3.1* fusion (pJNS03) and a P*_gapA_*::*dsRed2* expression construct (pFU228) were grown in LB medium at 25°C overnight. Mice were infected orally with 2×10^8^ bacteria. After five days mice were sacrificed by CO_2_ asphyxiation. For cryosections, the small intestine, colon, caecum, MLNs, spleen and liver were frozen in Tissue-Tek OCT freezing medium (Sakura Finetek) on dry ice. Sections of 8–10 mm were prepared using a Zeiss cryostat Hyrax C50 and mounted on SuperFrost Plus slides (Thermo Scientific). Air-dried sections were fixed for 10 min in ice-cold acetone and washed twice with PBS. For visualization of the nuclei in the fixed tissue, samples were stained with 49,6- diamidino-2-phenylindole (DAPI, Sigma) for 3–5 min, air-dried and mounted with 80% glycerol in PBS. Localization of yersiniae in the infected tissues and expression of the P*_cnfY_*::*gfpmut3.1* fusion of these bacteria were visualized by a fluorescence microscope (Axiovert II with Axiocam HR, Zeiss, Germany) using the AxioVision program (Zeiss, Germany).

To detect the *cnfY* gene expression during the infection process *Y. pseudotuberculosis* wild-type strain YPIII harboring the P*_cnfY_*::*luxCDABE* fusion vector pJNS02 or the empty vector pFU54 were grown in LB medium at 25°C overnight. About 2×10^8^ luminescent bacteria were used for oral infection. For *in vivo* imaging mice were anesthesized with isoflurane and the bacterial infection was followed daily using the IVIS Lumina system (Xenogen). To ensure maintenance of the plasmids during the course of infection, the bacteria were isolated from the small intestine, colon, caecum, MLNs, spleen and liver and tested for the presence of the plasmid.

### Histology

MLNs, spleen, liver, small intestine, caecum and colon were analyzed histopathologically of three mice per group. According to standard histology procedures, organs were fixed in 4% neutrally buffered formaldehyde for 24 to 48 h, embedded in paraffin and 3 µm sections were stained with hematoxylin-eosin (H & E). Sections were evaluated by light-microscopy blinded to the experimental groups.

## Supporting Information

Figure S1 *In vitro* expression analysis of the *cnfY-lacZ* fusion. *Y. pseudotuberculosis* YPIII pJNS04 (P*_cnfY_*::*lacZ*) was grown (**A**) in LB to exponential and stationary phase at 25°C and 37°C, or (**B**) in complex (LB, BHI) or minimal media (MMA, RPMI) at 37°C to stationary phase. The β-galactosidase activity of the cultures was determined from at least three independent cultures in triplicate. The asterisk indicate that there was a significant difference in the β-galactosidase activities based on an unpaired t-test. Stars indicate results that differed significantly from expression in LB medium *** (P<0.001).
(TIF)

Figure S2 Influence of *cnfY* on the bodyweight of BALB/c mice infected with *Y. pseudotuberculosis*. Body weight of BALB/c mice (n = 10/strain) were monitored up to 14 days after oral infection with 2×10^9^ cfu of *Y. pseudotuberculosis* YPIII (black line), the *cnfY* mutant YP147 (red line) harbouring the empty vector pJNS11, and the strains YPIII pJNS10 (*cnfY* ^+^) (green line) and YP147 pJNS10 (*cnfY* ^+^) (blue line).
(TIF)

Figure S3 Influence of *cnfY* on the survival of BALB/c mice infected with *phoP*+ *Y. pseudotuberculosis* YPIII derivatives. Survival (A) and body weight (B) of BALB/c mice (n = 10/strain) were monitored up to 14 days after oral infection with 2×10^9^ cfu of *Y. pseudotuberculosis* YP149 (black line) and the *cnfY* mutant YP180 (red line).
(TIF)

Figure S4 Influence of CNF_Y_ on weight of the organs and the gut length of BALB/c mice infected with *Y. pseudotuberculosis*. Weight of the spleen (A) and the liver (B) of BALB/c mice (n = 10/strain) were monitored up to seven days after oral infection with 2×10^8^ cfu of *Y. pseudotuberculosis* YPIII (black), the *cnfY* mutant YP147 (red). (C) Length of the intestine of BALB/c mice (n = 10/strain) were monitored at day six and seven after oral infection with 2×10^8^ cfu of *Y. pseudotuberculosis* YPIII (black), or the *cnfY* mutant YP147 (red). Stars indicate results of the organs infected with YP147 that differed significantly from those infected with YPIII with * (P<0.05), ** (P<0.01) and *** (P<0.001).
(TIF)

Figure S5 Gating strategies for the analysis of immune cells recruited to the spleen after infection with *Y. pseudotuberculosis* YPIII or YP147. Exemplary gating strategy of splenocytes from YP147 (Δ*cnfy*)-infected mice at day three post infection. (**A**) T cells = CD19^−^CD3^+^, B cells = CD19^+^CD3^−^, natural killer (NK) cells = CD19^−^CD3^−^NKp46^+^. (**B**) Neutrophils = CD49b^−^CD19^−^CD3^−^Ly-6G^+^CD11b^+^. dendritic cells (DCs) = CD49b^−^CD19^−^CD3^−^Ly-6G^−^CD11b^−^Ly-6C^−^CD11c^+^, macrophages/monocytes = CD49b^−^CD19^−^CD3^−^Ly-6G^−^CD11c^−^CD11b^+^.
(TIF)

Figure S6 Analysis of immune cells recruited to the spleen after infection with *Y. pseudotuberculosis* YPIII or YP147. About 2×10^8^ bacteria (YPIII, YP147) were used to infect BALB/c mice. Three days after infection, mice were sacrificed, the spleens were isolated, homogenized and the cell suspensions were used for flow cytometric analysis. Values on the y axis indicate the numbers of cells isolated from spleen infected with the wild-type strain YPIII or the *cnfY* mutant strain YP147. CD11b^+^/Ly6G^−^: macrophages/monocytes; CD11b^+^/Ly6G^+^: neutrophils; CD11c^+^: DCs; NKp46^+^: NK cells, CD3^+^: T cells, CD19^+^: B cells. The data show the median from at least two different experiments each done with groups of 4–6 mice. The asteriks indicates that there was a significant difference in the number of the indicated cells type in the whole organ based on a Mann-Whitney test. Stars indicate results that differed significantly from those of YPIII with * (P<0.05), ** (P<0.01) and *** (P<0.001).
(TIF)

Figure S7 Effect of YopE on CNF_Y_-induced Rho GTPase activation in macrophages. Murine macrophages were either incubated for 3 h with 25 nM purified CNF_Y_ or left untreated. After incubation, cells were infected with *Y. pseudotuberculosis* YPIII (wild-type) or the *yopE* mutant YP275 grown at 37°C with a MOI of 100 from a culture grown at 37°C. Cells were lysed and aliquots of the cell lysates were taken for westernblot analysis. (**A**) Remaining lysates were incubated with beads, coupled with GTPase-binding domains of signaling molecules interacting with GTP-bound RhoA or Rac1/Cdc42. The total and activated amounts of the RhoA and Rac1 GTPases in the lysates were analyzed with specific antibodies. (**B**) Intracellular Yops were visualized using an antiserum directed against all secreted Yops (α-Yop). The size of the molecular marker (kDa) is given on the left. Strain YP101 (Δ*ycsS*) was used as negative control to rule out permeabilization of the membrane in the detergent solubility assay.
(TIF)

Figure S8 Absence of CNF_Y_ reduces Yop delivery into primary cells of MLNs. Single cell suspension of MLNs of six 8-week-old BALB/c mice were prepared and infected with YPIII-ETEM (YP173) and YP147 Δ*cnfY*-ETEM (YP217) at an MOI of 10 for 1 h. YPIII and YP174 was used as negative controls. Two independent experiments were performed each done with groups of three mice. The percentage of blue cells of the suspensions was plotted and the median is presented. The asteriks indicate that percentage of blue cells differed significantly in the organ based on a Mann-Whitney test. Stars indicate results of YP147 that differed significantly from those of YPIII with ** (P<0.01).
(TIF)

Figure S9 Gating strategies for the analysis of CNF_Y_ on Yop delivery. Exemplary gating strategies of MLNs cells of YPIII-ETEM infected mice at day three post infection. *Ex vivo* cells were subjected to CCF4-AM treatment. Alive cells are “green”, translocated cells are “blue”. (**A**) T cells = CD19^−^CD3^+^, B cells = CD19^+^CD3^−^, Neutrophils = CD19^−^CD3^−^Ly-6G^+^CD11b^+^, dendritic cells (DCs) = CD19^−^CD3^−^CD11c^+^, Macrophages/Monocytes = CD49b^−^CD19^−^CD3^−^Ly-6G^−^CD11b^+^. (B) Natural killer (NK) cells = CD19^−^CD3^−^NKp46^+^.
(TIF)

Figure S10 Absence of CNF_Y_ reduces Yop delivery into host cells in PPs, MLNs and spleen during infection. (**A**) BALB/c mice were orally infected with 2×10^9^ cfu of YPIII-ETEM (YP173) and YP147 Δ*cnfY*-ETEM (YP217). YPIII and YP101 Δ*yscS*-ETEM (YP174) were used as negative controls. Day three post infection the MLNs, liver and spleen were isolated and filtred to generate single-cell suspensions. Cells were labeled with antibodies to the indicated surface markers for macrophages, dendritic cells (DCs), neutrophils, natural killer (NK) cells, B and T cells and incubated with CCF4-AM. The percentage of blue cells was analyzed by flow cytometry. (**A**) Detection of green and blue cells by flow cytometry in PP, MLNs and spleen of mice infected with YP173 and YP217. The percentage of blue cells among all living cells are plotted. (**B**) The percentage of blue cells among identified cell types of the MLNs and the spleen are plotted. The experiment was repeated three times with groups of 4–6 mice. The asterisks indicate that percentage of blue cells in YP147-infected mice differed significantly from those infected with YPIII; * (P<0.05), ** (P<0.01) and *** (P<0.001).
(TIF)
