# Supplementary figures and images for: The Cytotoxic Necrotizing Factor of Yersinia pseudotuberculosis (CNFY) Enhances Inflammation and Yop Delivery during Infection by Activation of Rho GTPases
Source: PLoS Pathog. 2013 Nov 7;9(11):e1003746. doi: 10.1371/journal.ppat.1003746 (PMC3820761; doi:10.1371/journal.ppat.1003746)

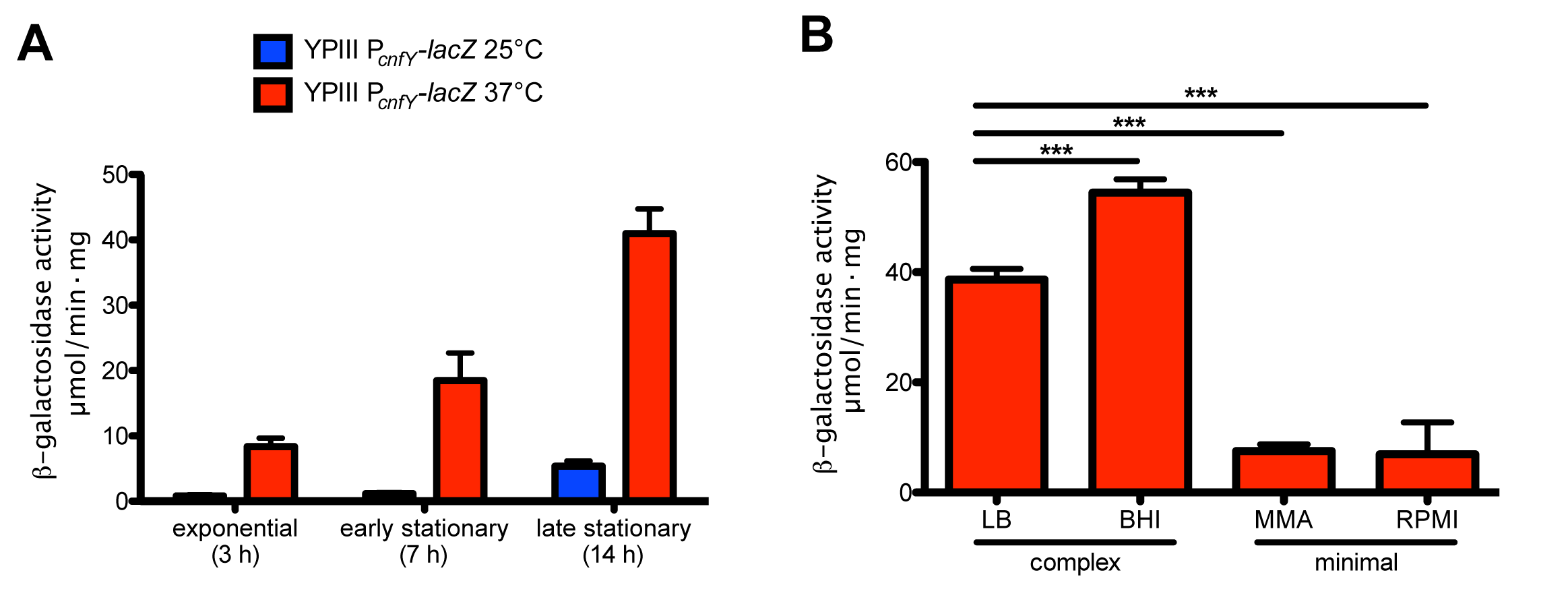

Supplement: Figure S1 — In vitro expression analysis of the cnfY-lacZ fusion. Y. pseudotuberculosis YPIII pJNS04 (PcnfY::lacZ) was grown (A) in LB to exponential and stationary phase at 25°C and 37°C, or (B) in complex (LB, BHI) or minimal media (MMA, RPMI) at 37°C to stationary phase. The β-galactosidase activity of the cultures was determined from at least three independent cultures in triplicate. The asterisk indicate that there was a significant difference in the β-galactosidase activities based on an unpaired t-test. Stars indicate results that differed significantly from expression in LB medium *** (P<0.001). (TIF) [file ppat.1003746.s001.tif]

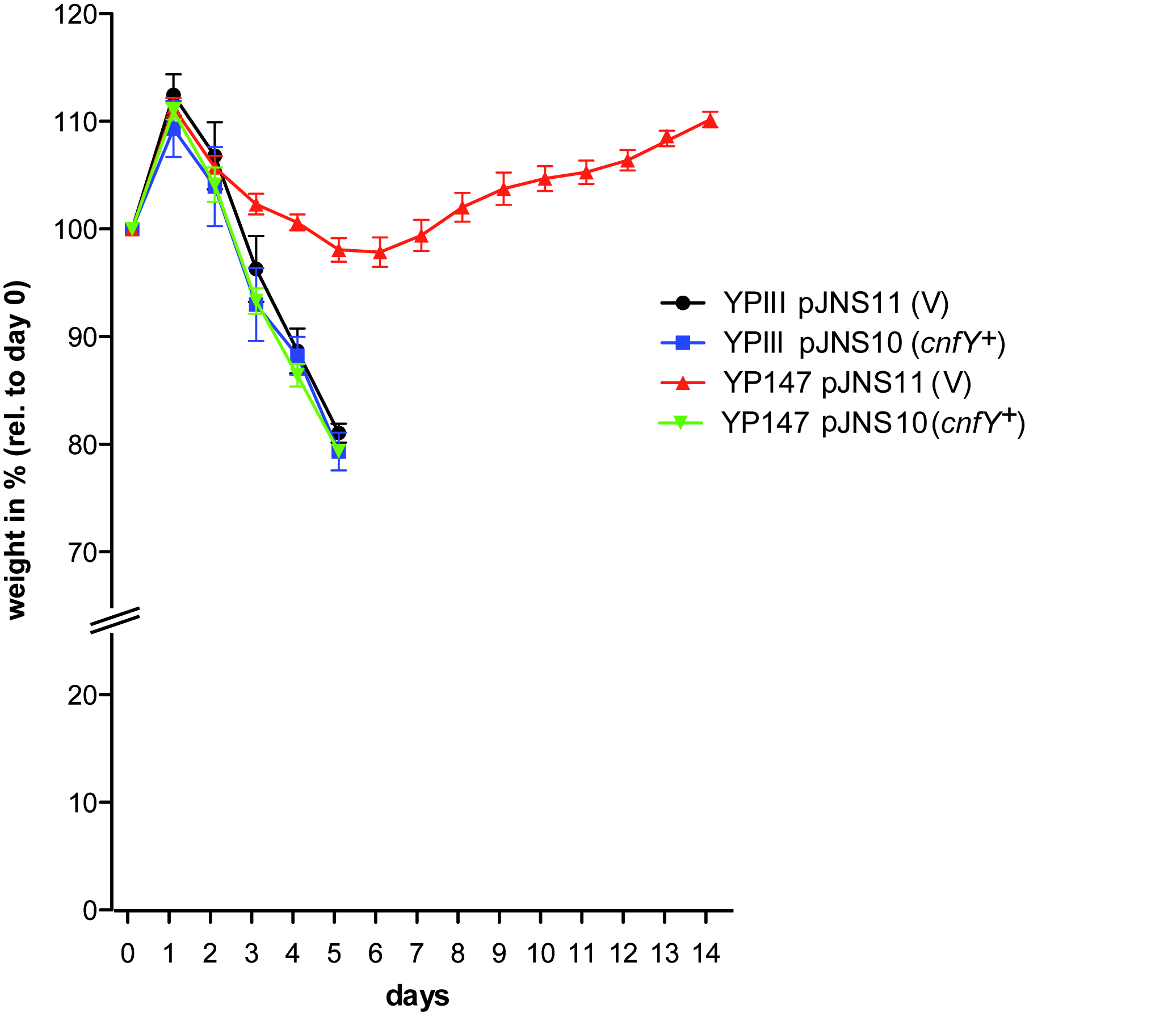

Supplement: Figure S2 — Influence of cnfY on the bodyweight of BALB/c mice infected with Y. pseudotuberculosis. Body weight of BALB/c mice (n = 10/strain) were monitored up to 14 days after oral infection with 2×10^9^ cfu of Y. pseudotuberculosis YPIII (black line), the cnfY mutant YP147 (red line) harbouring the empty vector pJNS11, and the strains YPIII pJNS10 (cnfY +) (green line) and YP147 pJNS10 (cnfY +) (blue line). (TIF) [file ppat.1003746.s002.tif]

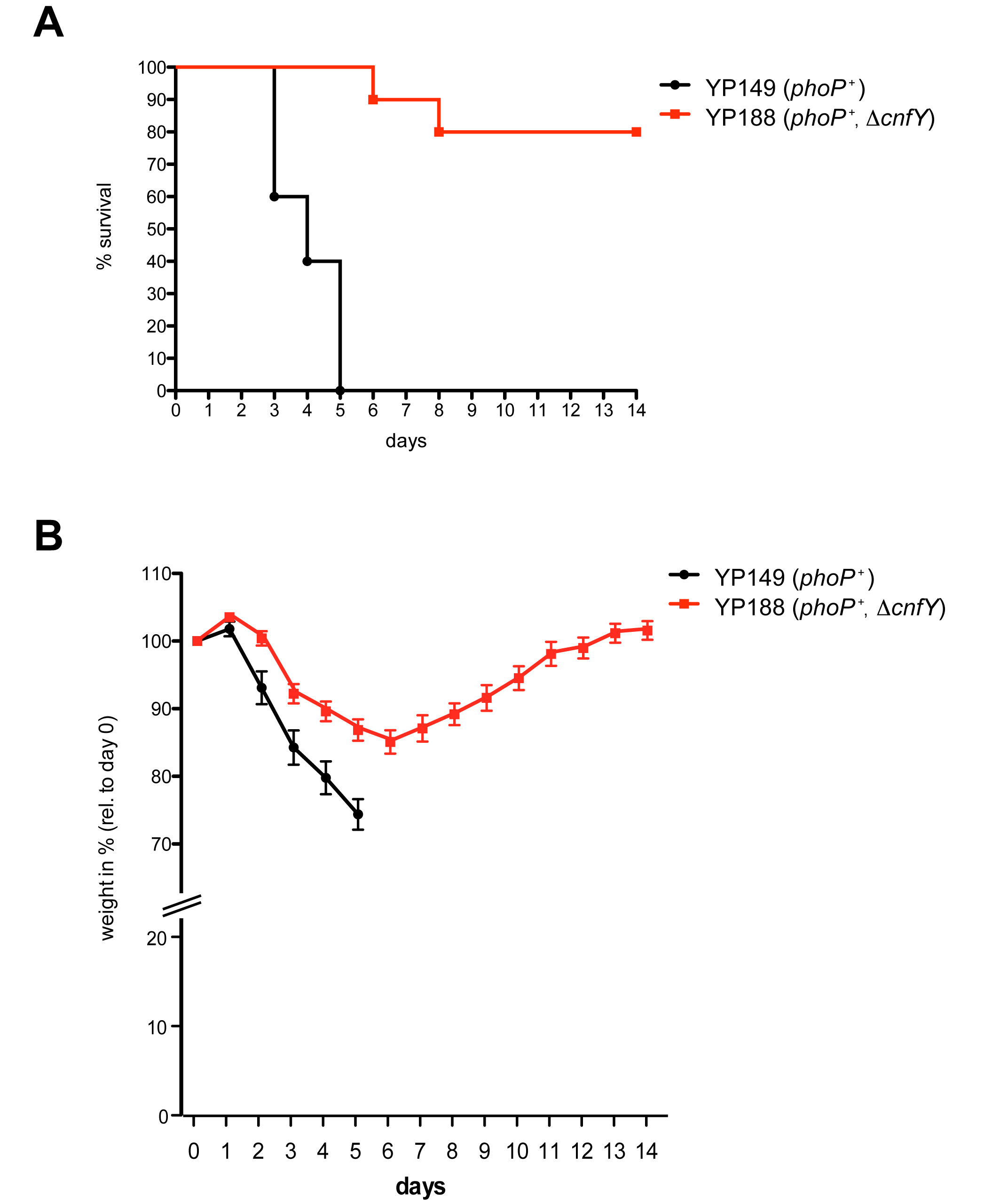

Supplement: Figure S3 — Influence of cnfY on the survival of BALB/c mice infected with phoP+ Y. pseudotuberculosis YPIII derivatives. Survival (A) and body weight (B) of BALB/c mice (n = 10/strain) were monitored up to 14 days after oral infection with 2×10^9^ cfu of Y. pseudotuberculosis YP149 (black line) and the cnfY mutant YP180 (red line). (TIF) [file ppat.1003746.s003.tif]

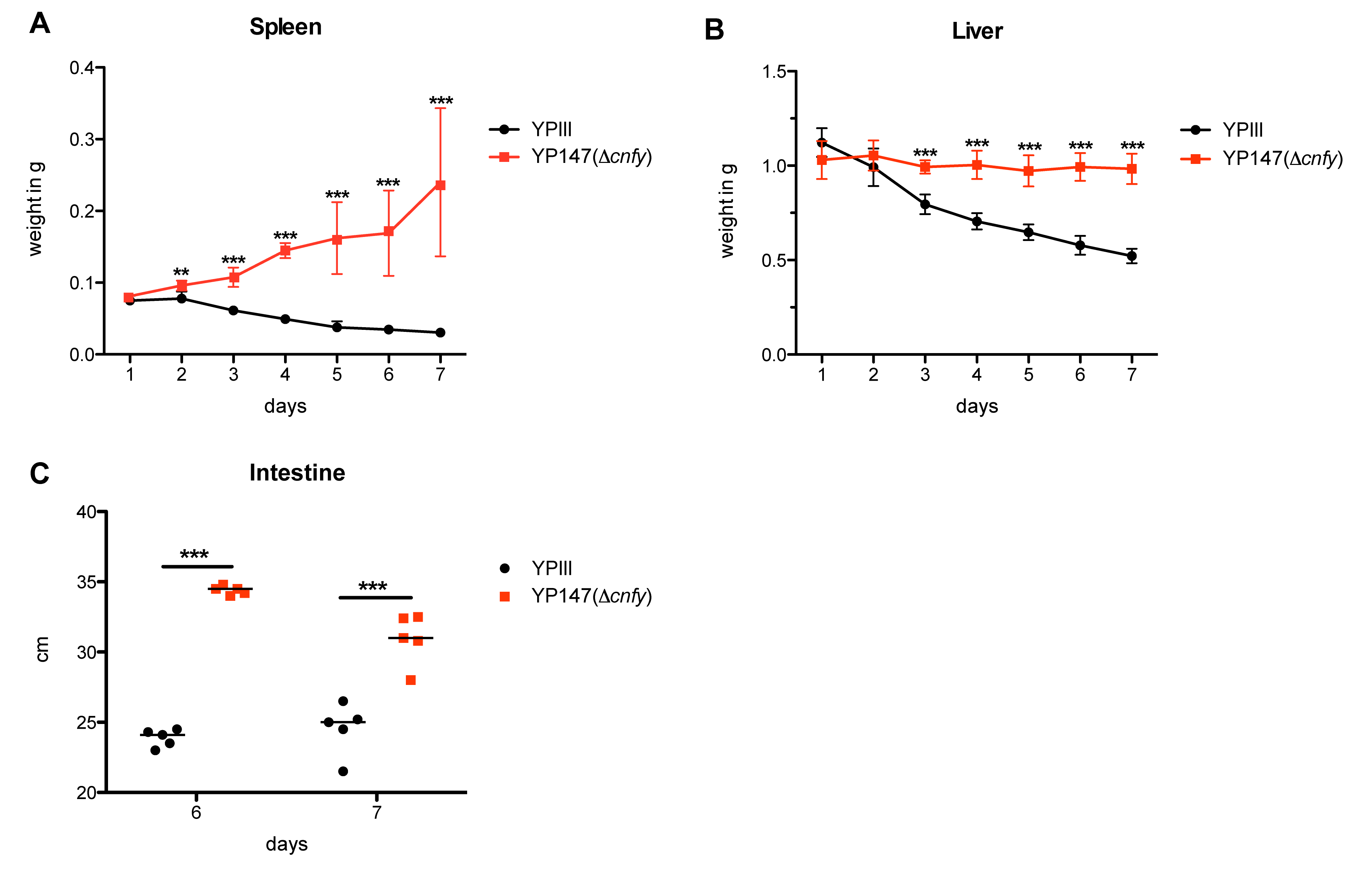

Supplement: Figure S4 — Influence of CNFY on weight of the organs and the gut length of BALB/c mice infected with Y. pseudotuberculosis. Weight of the spleen (A) and the liver (B) of BALB/c mice (n = 10/strain) were monitored up to seven days after oral infection with 2×10^8^ cfu of Y. pseudotuberculosis YPIII (black), the cnfY mutant YP147 (red). (C) Length of the intestine of BALB/c mice (n = 10/strain) were monitored at day six and seven after oral infection with 2×10^8^ cfu of Y. pseudotuberculosis YPIII (black), or the cnfY mutant YP147 (red). Stars indicate results of the organs infected with YP147 that differed significantly from those infected with YPIII with * (P<0.05), ** (P<0.01) and *** (P<0.001). (TIF) [file ppat.1003746.s004.tif]

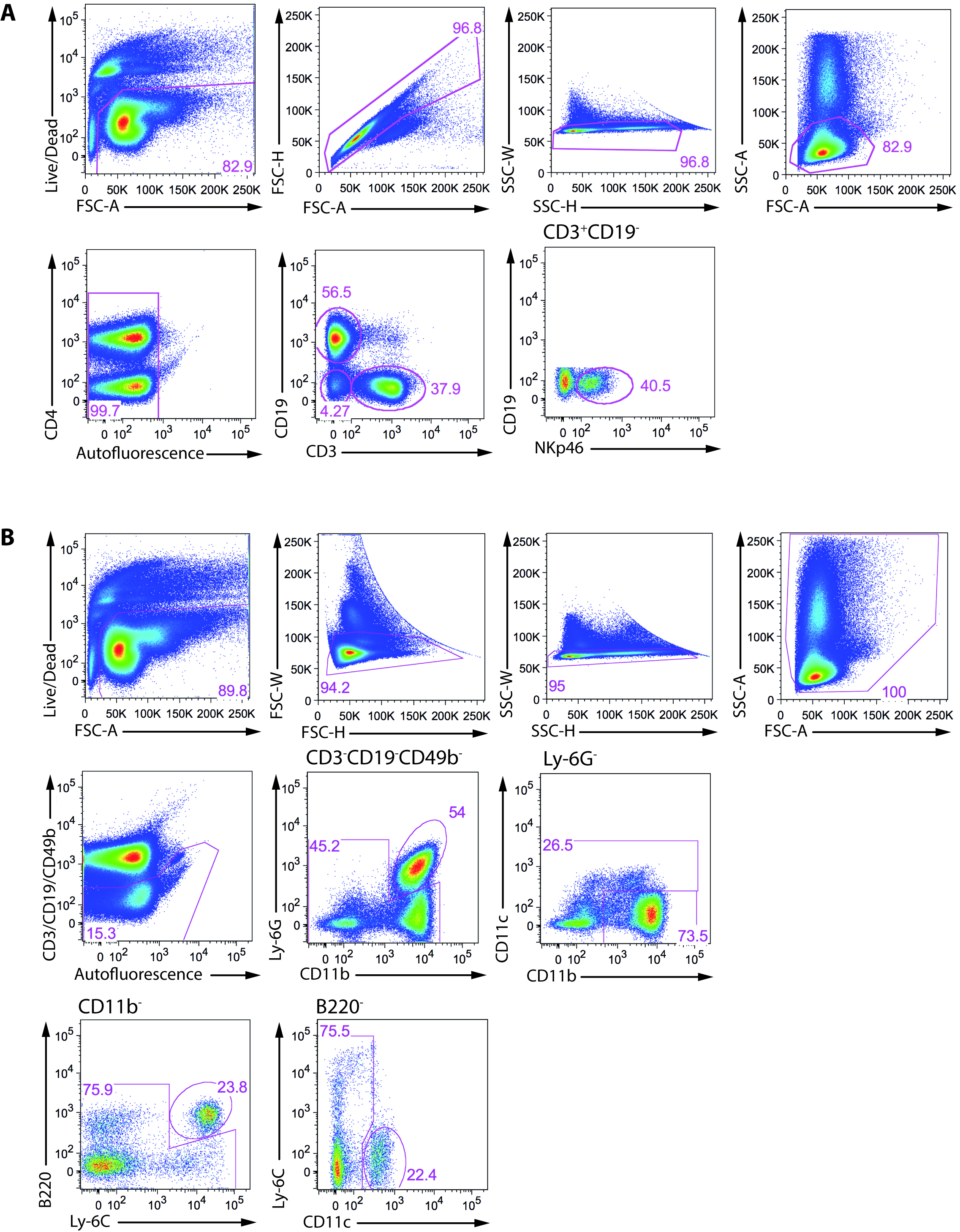

Supplement: Figure S5 — Gating strategies for the analysis of immune cells recruited to the spleen after infection with Y. pseudotuberculosis YPIII or YP147. Exemplary gating strategy of splenocytes from YP147 (Δcnfy)-infected mice at day three post infection. (A) T cells = CD19−CD3+, B cells = CD19+CD3−, natural killer (NK) cells = CD19−CD3−NKp46+. (B) Neutrophils = CD49b−CD19−CD3−Ly-6G+CD11b+. dendritic cells (DCs) = CD49b−CD19−CD3−Ly-6G−CD11b−Ly-6C−CD11c+, macrophages/monocytes = CD49b−CD19−CD3−Ly-6G−CD11c−CD11b+. (TIF) [file ppat.1003746.s005.tif]

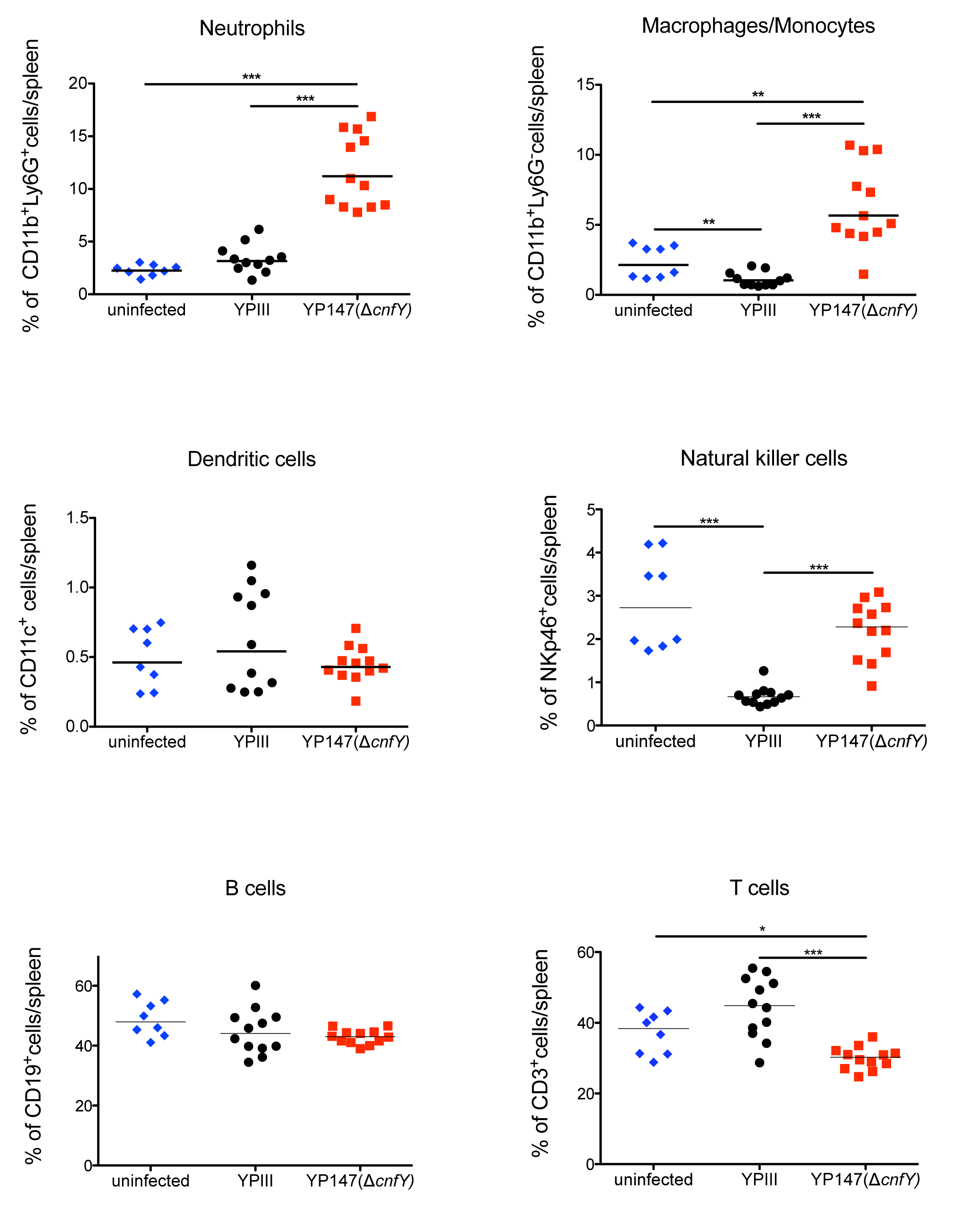

Supplement: Figure S6 — Analysis of immune cells recruited to the spleen after infection with Y. pseudotuberculosis YPIII or YP147. About 2×10^8^ bacteria (YPIII, YP147) were used to infect BALB/c mice. Three days after infection, mice were sacrificed, the spleens were isolated, homogenized and the cell suspensions were used for flow cytometric analysis. Values on the y axis indicate the numbers of cells isolated from spleen infected with the wild-type strain YPIII or the cnfY mutant strain YP147. CD11b+/Ly6G−: macrophages/monocytes; CD11b+/Ly6G+: neutrophils; CD11c+: DCs; NKp46+: NK cells, CD3+: T cells, CD19+: B cells. The data show the median from at least two different experiments each done with groups of 4–6 mice. The asteriks indicates that there was a significant difference in the number of the indicated cells type in the whole organ based on a Mann-Whitney test. Stars indicate results that differed significantly from those of YPIII with * (P<0.05), ** (P<0.01) and *** (P<0.001). (TIF) [file ppat.1003746.s006.tif]

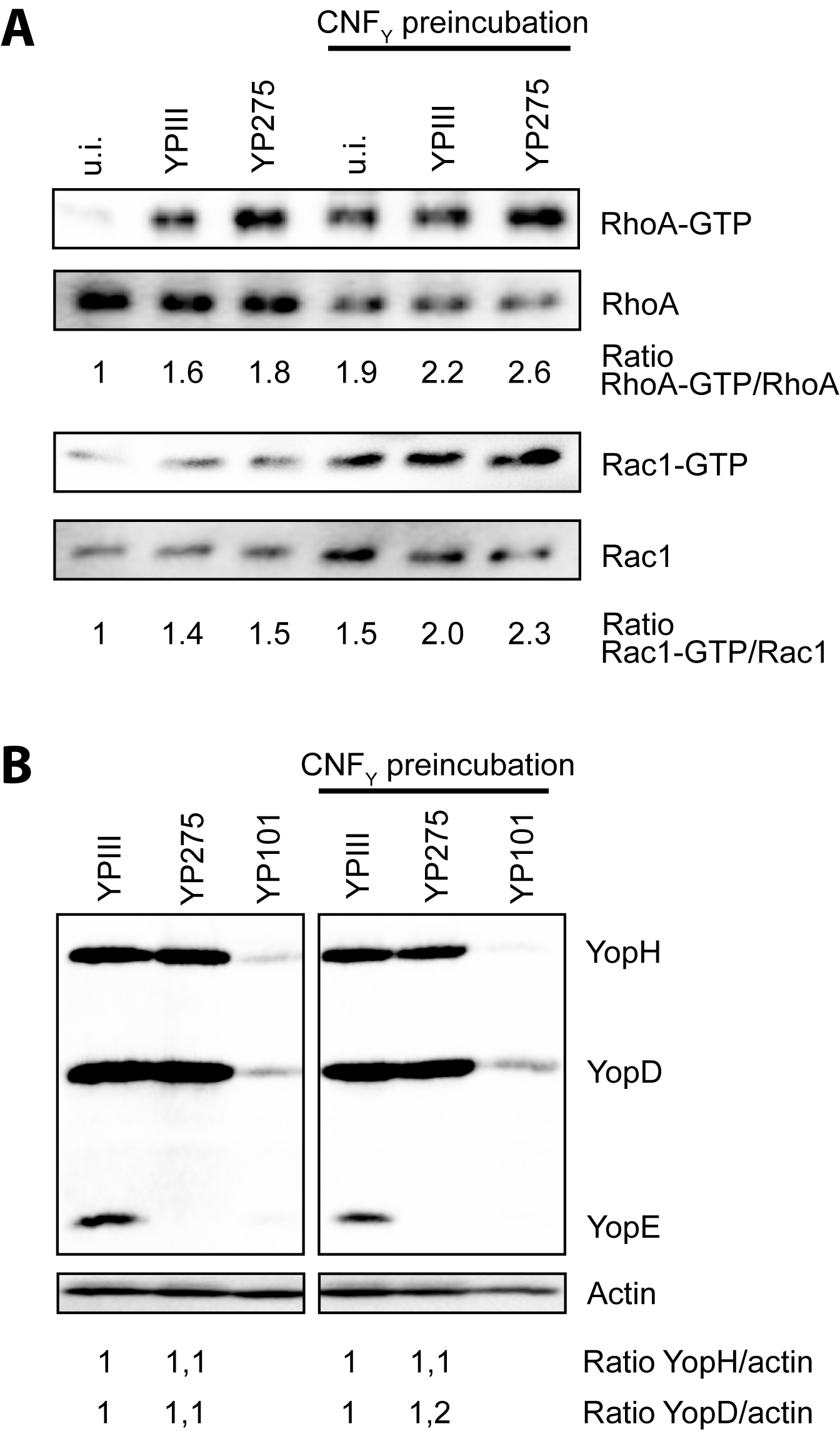

Supplement: Figure S7 — Effect of YopE on CNFY-induced Rho GTPase activation in macrophages. Murine macrophages were either incubated for 3 h with 25 nM purified CNFY or left untreated. After incubation, cells were infected with Y. pseudotuberculosis YPIII (wild-type) or the yopE mutant YP275 grown at 37°C with a MOI of 100 from a culture grown at 37°C. Cells were lysed and aliquots of the cell lysates were taken for westernblot analysis. (A) Remaining lysates were incubated with beads, coupled with GTPase-binding domains of signaling molecules interacting with GTP-bound RhoA or Rac1/Cdc42. The total and activated amounts of the RhoA and Rac1 GTPases in the lysates were analyzed with specific antibodies. (B) Intracellular Yops were visualized using an antiserum directed against all secreted Yops (α-Yop). The size of the molecular marker (kDa) is given on the left. Strain YP101 (ΔycsS) was used as negative control to rule out permeabilization of the membrane in the detergent solubility assay. (TIF) [file ppat.1003746.s007.tif]

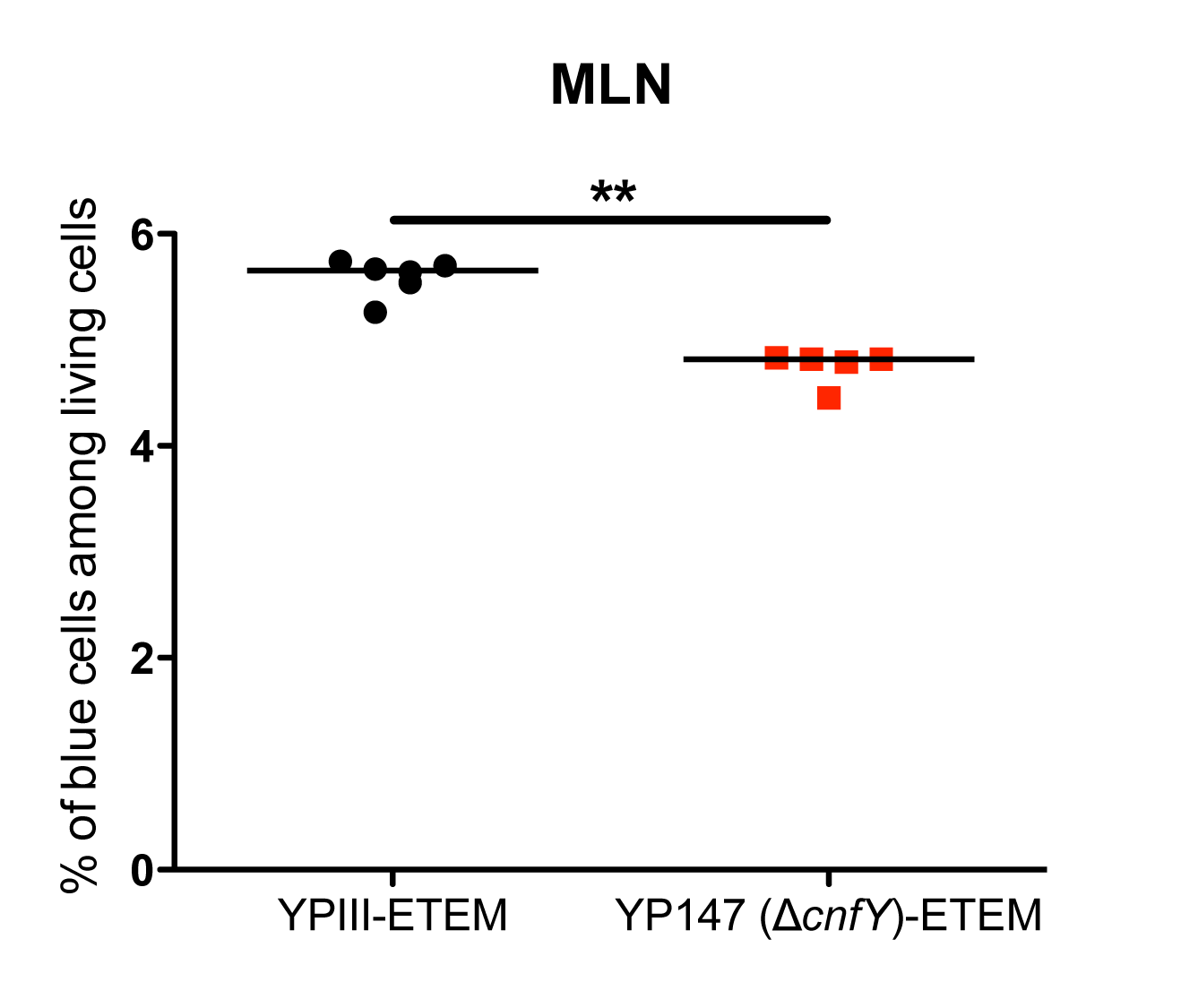

Supplement: Figure S8 — Absence of CNFY reduces Yop delivery into primary cells of MLNs. Single cell suspension of MLNs of six 8-week-old BALB/c mice were prepared and infected with YPIII-ETEM (YP173) and YP147 ΔcnfY-ETEM (YP217) at an MOI of 10 for 1 h. YPIII and YP174 was used as negative controls. Two independent experiments were performed each done with groups of three mice. The percentage of blue cells of the suspensions was plotted and the median is presented. The asteriks indicate that percentage of blue cells differed significantly in the organ based on a Mann-Whitney test. Stars indicate results of YP147 that differed significantly from those of YPIII with ** (P<0.01). (TIF) [file ppat.1003746.s008.tif]

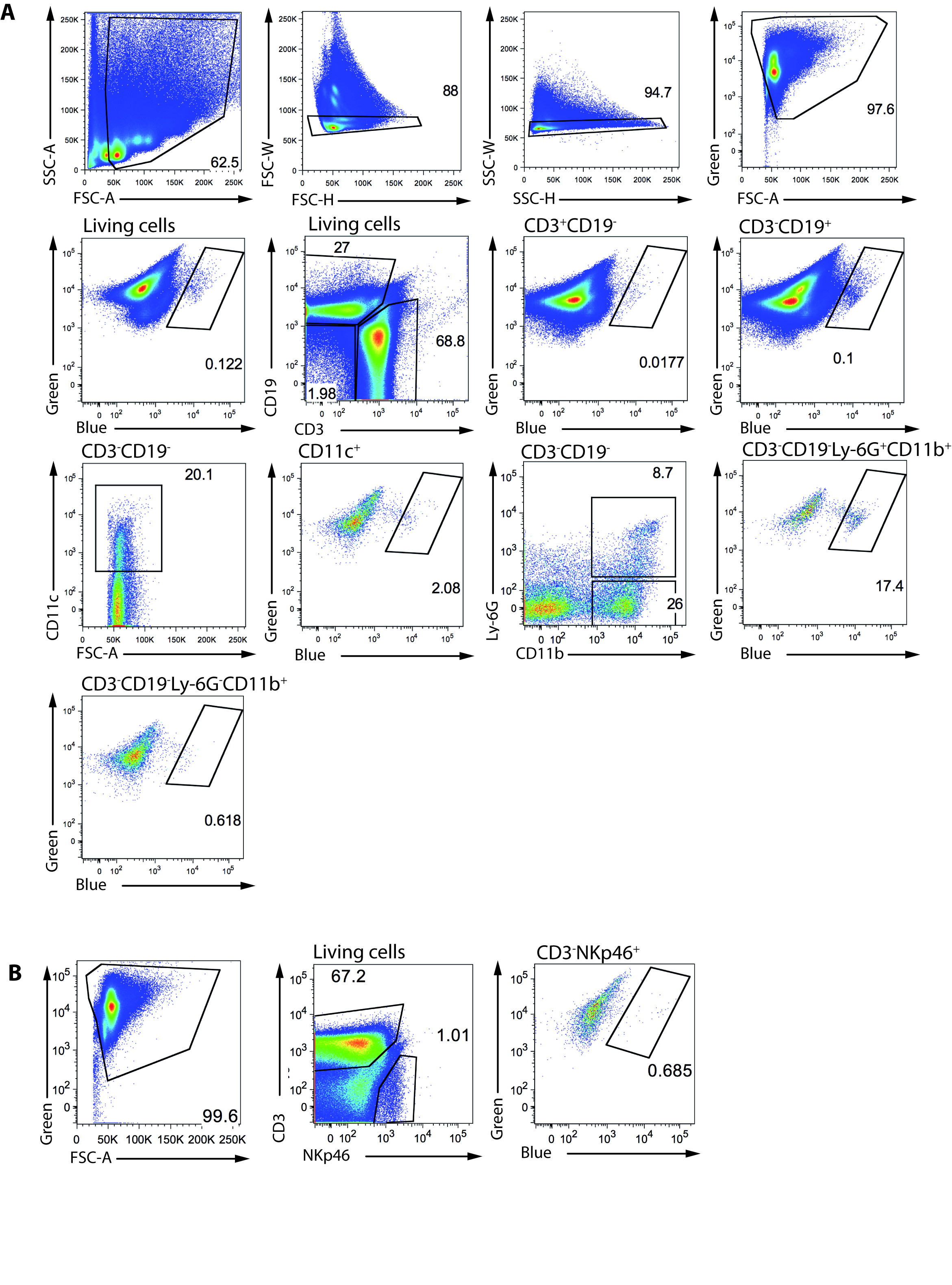

Supplement: Figure S9 — Gating strategies for the analysis of CNFY on Yop delivery. Exemplary gating strategies of MLNs cells of YPIII-ETEM infected mice at day three post infection. Ex vivo cells were subjected to CCF4-AM treatment. Alive cells are “green”, translocated cells are “blue”. (A) T cells = CD19−CD3+, B cells = CD19+CD3−, Neutrophils = CD19−CD3−Ly-6G+CD11b+, dendritic cells (DCs) = CD19−CD3−CD11c+, Macrophages/Monocytes = CD49b−CD19−CD3−Ly-6G−CD11b+. (B) Natural killer (NK) cells = CD19−CD3−NKp46+. (TIF) [file ppat.1003746.s009.tif]

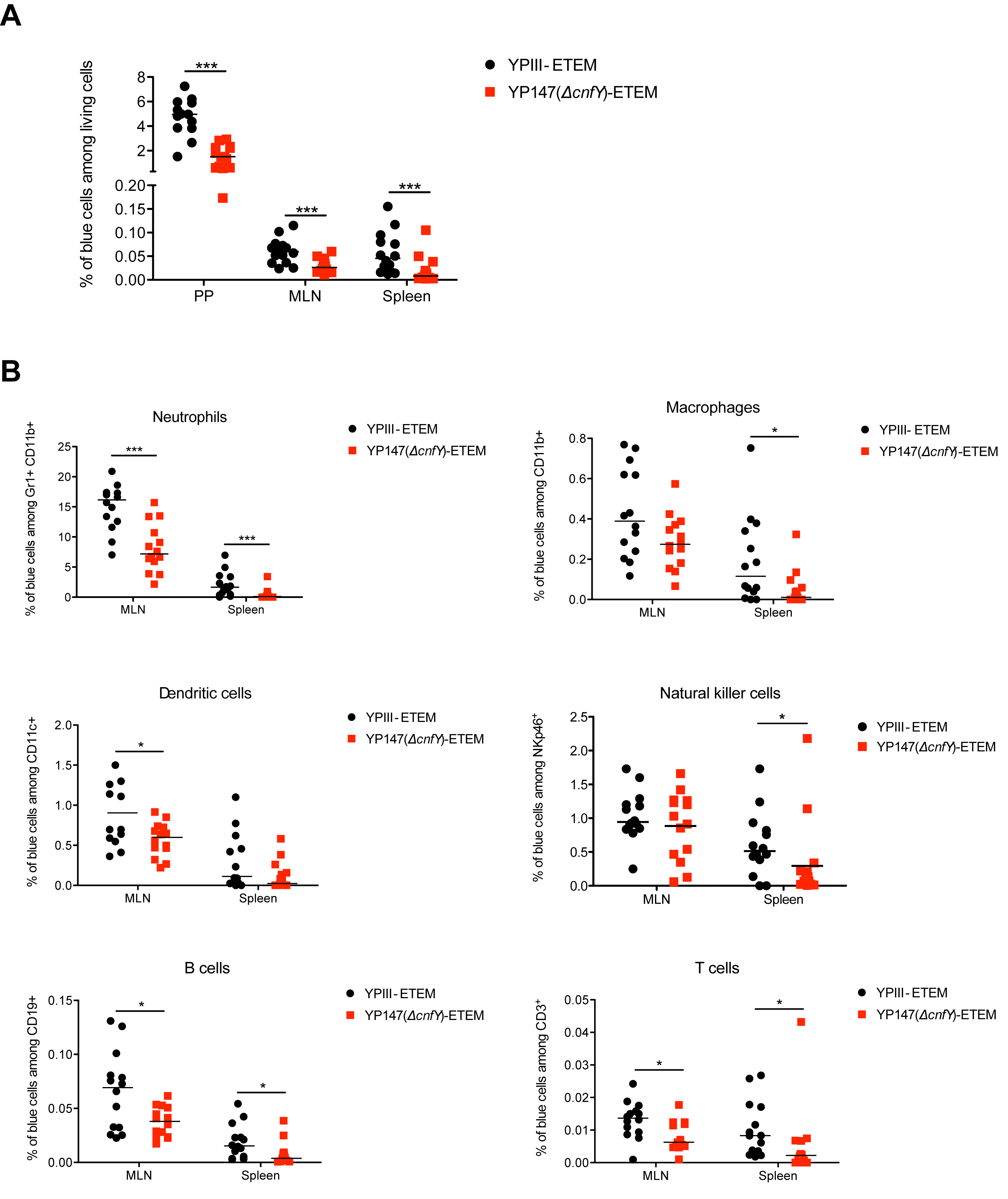

Supplement: Figure S10 — Absence of CNFY reduces Yop delivery into host cells in PPs, MLNs and spleen during infection. (A) BALB/c mice were orally infected with 2×10^9^ cfu of YPIII-ETEM (YP173) and YP147 ΔcnfY-ETEM (YP217). YPIII and YP101 ΔyscS-ETEM (YP174) were used as negative controls. Day three post infection the MLNs, liver and spleen were isolated and filtred to generate single-cell suspensions. Cells were labeled with antibodies to the indicated surface markers for macrophages, dendritic cells (DCs), neutrophils, natural killer (NK) cells, B and T cells and incubated with CCF4-AM. The percentage of blue cells was analyzed by flow cytometry. (A) Detection of green and blue cells by flow cytometry in PP, MLNs and spleen of mice infected with YP173 and YP217. The percentage of blue cells among all living cells are plotted. (B) The percentage of blue cells among identified cell types of the MLNs and the spleen are plotted. The experiment was repeated three times with groups of 4–6 mice. The asterisks indicate that percentage of blue cells in YP147-infected mice differed significantly from those infected with YPIII; * (P<0.05), ** (P<0.01) and *** (P<0.001). (TIF) [file ppat.1003746.s010.tif]
